# Manganese porphyrin-based metal-organic framework for synergistic sonodynamic therapy and ferroptosis in hypoxic tumors

**DOI:** 10.7150/thno.45511

**Published:** 2021-01-01

**Authors:** Qingbo Xu, Guiting Zhan, Zelong Zhang, Tuying Yong, Xiangliang Yang, Lu Gan

**Affiliations:** 1 National Engineering Research Center for Nanomedicine, College of Life Science and Technology, Huazhong University of Science and Technology, Wuhan 430074, China.; 2 Zhuhai Precision Medical Center, Zhuhai People's Hospital (Zhuhai Hospital Affiliated with Jinan University), Zhuhai 519000, China.; 3 Hubei Key Laboratory of Bioinorganic Chemistry and Materia Medica, School of Chemistry and Chemical Engineering, Huazhong University of Science and Technology, Wuhan 430074, China.; 4 Key Laboratory of Molecular Biophysics of Ministry of Education, College of Life Science and Technology, Huazhong University of Science and Technology, Wuhan 430074, China.

**Keywords:** metal-organic framework, catalase-like activity, GSH depletion, reactive oxygen species, sonodynamic therapy, ferroptosis

## Abstract

Development of efficient therapeutic strategy to incorporate ultrasound (US)-triggered sonodynamic therapy (SDT) and ferroptosis is highly promising in cancer therapy. However, the SDT efficacy is severely limited by the hypoxia and high glutathione (GSH) in the tumor microenvironment, and ferroptosis is highly associated with reactive oxygen species (ROS) and GSH depletion.

**Methods:** A manganese porphyrin-based metal-organic framework (Mn-MOF) was constructed as a nanosensitizer to self-supply oxygen (O_2_) and decrease GSH for enhanced SDT and ferroptosis. *In vitro* and *in vivo* analysis, including characterization, O_2_ generation, GSH depletion, ROS generation, lipid peroxidation, antitumor efficacy and tumor immune microenvironment were systematically evaluated.

**Results:** Mn-MOF exhibited catalase-like and GSH decreasing activity *in vitro*. After efficient internalization into cancer cells, Mn-MOF persistently catalyzed tumor-overexpressed H_2_O_2_ to *in-situ* produce O_2_ to relieve tumor hypoxia and decrease GSH and GPX4, which facilitated the formation of ROS and ferroptosis to kill cancer cells upon US irradiation in hypoxic tumors. Thus, strong anticancer and anti-metastatic activity was found in H22 and 4T1 tumor-bearing mice after a single administration of Mn-MOF upon a single US irradiation. In addition, Mn-MOF showed strong antitumor immunity and improved immunosuppressive microenvironment upon US irradiation by increasing the numbers of activated CD8^+^ T cells and matured dendritic cells and decreaing the numbers of myeloid-derived suppressor cells in tumor tissues.

**Conclusions:** Mn-MOF holds great potential for hypoxic cancer therapy.

## Introduction

Ultrasound (US)-triggered sonodynamic therapy (SDT), as a non-invasive therapeutic modality, has attracted considerable attention for cancer treatment 1,2. SDT combines a sonosensitizer, US source and oxygen (O_2_) to generate reactive oxygen species (ROS) to kill cancer cells, which can break through the barrier of low tissue penetrating depth of photodynamic therapy (PDT) 3. Meanwhile, SDT shows tremendous potential to induce immunogenic cell death (ICD) and activate antitumor immunity. However, tumor microenvironment, such as hypoxia and high glutathione (GSH), severely influences the therapeutic effects of SDT 4,5. Therefore, it is highly desirable to develop a suitable sonosensitizer with the self-sufficiency of O_2_ and GSH decreasing capacity to synergistically increase ROS levels, improving the therapeutic effects of SDT and enhancing antitumor immunity.

Ferroptosis, an iron-dependent form of regulated cell death, has received great attention in cancer treatment 6. Ferroptosis is characterized by the accumulation of ROS and lipid peroxidation (LPO) products to lethal levels 7-9, which efficiently eliminates tumor cells. However, as a central downstream regulator of ferroptosis, selenoenzyme glutathione peroxidase 4 (GPX4) combats with LPO by using two molecules of GSH as electron donors to reduce toxic phospholipid hydroperoxides into nontoxic phospholipid alcohols 10. Various GPX4-inactivating agents, including direct GPX4 inhibitors 10 and GSH scavengers 11, which indirectly cause the loss of GPX4 activity, can induce ferroptotic cell death. Therefore, the development of efficient SDT therapy with simultaneous GPX4 depletion might induce ferroptosis, which incorporates as a highly promising therapeutic approach in cancer therapy.

O_2_ self-produced nanoplatforms, such as manganese dioxide (MnO_2_) nanoparticles and catalase-loaded nanoparticles triggered by unique tumor microenvironment (i. e, high concentration of H_2_O_2_), have been reported to relieve hypoxia to attenuate the ROS-based therapies 12,13. However, the rapid consumption of MnO_2_ and the low stability and easy inactivation of natural catalase cannot continuously catalyze H_2_O_2_ into O_2_ 14,15, thus only alleviating hypoxia temporally. Meanwhile, these nanoparticles are difficult to simultaneously realize the O_2_ supply and intracellular GSH reduction 16,17. Manganese complexes have a catalytic reactivity to decompose H_2_O_2_ to generate O_2_ 18. Manganese porphyrins, the most widely used sonosensitizers in SDT, exhibit catalase-like activity due to their extensive conjugated ring system and stable redox activity that can undergo reversible one-electron transfers 19. During the decomposition of H_2_O_2_ to generate O_2_, Mn^3+^-porphyrins react with H_2_O_2_ to yield Mn^4+^-porphyrins, which react with another H_2_O_2_ to return back to the Mn^3+^-porphyrins, showing persistent catalytic ability of H_2_O_2_ 20. However, the hydrophobic nature, insufficient tumor localization and fast metabolism of manganese porphyrins restrict the SDT efficacy 21,22.

**Scheme 1:**
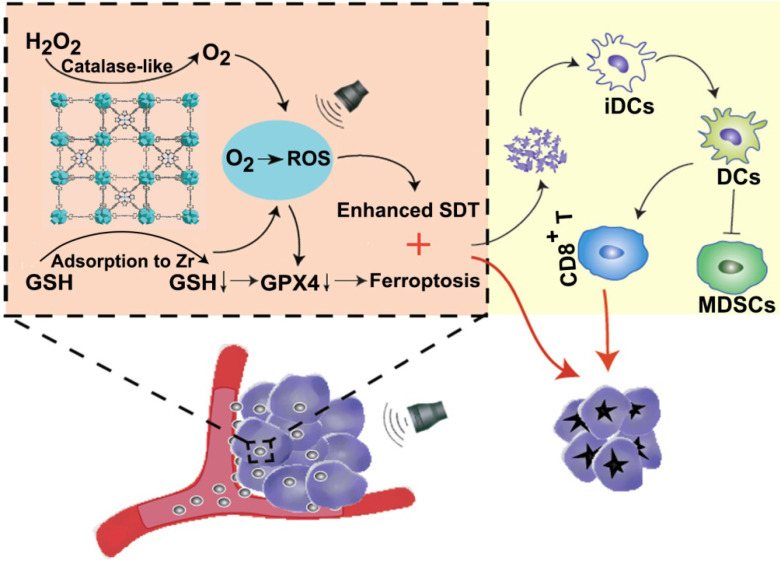
Schematic illustration of Mn-MOF for enhanced SDT and ferroptosis in cancer therapy. Mn-MOF generates ROS for ehanced SDT by catalase-like catalysis of H_2_O_2_ to O_2_ and GSH decrease in tumor cells upon US irradiation. Meanwhile, ROS and the decreased GPX4 activity induce ferroptosis in tumor cells. The combined SDT and ferroptosis generate strong antitumor immunity.

Nanoscale metal-organic frameworks (MOFs), a class of hybrid crystalline porous nanomaterials consisting of metal ions or clusters coordinated to organic ligands, have been widely used for biomedical applications 23,24. Porphyrin-based MOFs are designed for highly effective photosensitizers (PSs) in PDT due to high PS loading capacity without self-quenching 25,26. The porous structures and large surface area of MOFs also accelerate the diffusion of ROS, improving the PDT efficacy of MOFs 27. Here, we constructed a manganese porphyrin-based MOF (Mn-MOF) for SDT and ferroptosis in tumors by using the biocompatible metal ion zirconium (Zr) 28,29 as the joints, bound by manganese 5,10,15,20-tetrakis (4-benzoic acid) porphyrin (Mn-TCPP) as a sonosensitive bridging ligand (**Scheme 1**). Mn-TCPP with catalase-like activity induced the decomposition of H_2_O_2_ to persistently produce O_2_ at the hypoxic tumor site, which can overcome hypoxia and enhance the generation of Mn-MOF-induced ROS under US irradiation. Meanwhile, Zr decreased intracellular GSH to further increase the Mn-MOF-induced ROS level upon US irradiation, thus decreasing GPX4 activity and enhancing ROS-generated ferroptosis. The multiple functions of Mn-MOF combined to substantially improve the SDT and ferroptosis efficiency, which generated strong anticancer activity in both subcutaneous and metastatic tumor models. Meanwhile, Mn-MOF significantly improved tumor immune microenvironment by increasing the numbers of activated CD8^+^ T cells and matured dendritic cells and decreaing the numbers of myeloid-derived suppressor cells (MDSCs) in tumor tissues. Our findings show that Mn-MOF holds great potential to serve as an efficient agent for hypoxic cancer therapy.

## Results and Discussion

### Synthesis and characterization of Mn-MOF

To confirm the catalase-like catalytic role of Mn-TCPP in Mn-MOF, we synthesized Mn-MOF and MOF, in which Mn-TCPP and TCPP was used as the sonosensitive bridging ligand, respectively. The organic ligands Mn-TCPP and TCPP were first synthesized as depicted in **Figure S1**. Briefly, 5,10,15,20-tetrakis (4-methoxycarbonylphenyl) porphyrin (H_2_TCPP-OMe) was first synthesized by reflux reaction of 4-carbomethoxybenzaldehyde with pyrrole in propionic acid. Mn-TCPP or TCPP was obtained by reaction of H_2_TCPP-OMe with or without MnCl_2_·4H_2_O and then demethylation in KOH solution. Mn-TCPP and TCPP were characterized by Fourier transform infrared spectroscopy (FT-IR) (**Figure S2**), in which the vibration peak of N-Mn bond at about 1010 cm^-1^ indicated that Mn complexed to porphyrin ring in Mn-TCPP. ^1^H nuclear magnetic resonance (NMR) spectrum confirmed the chemical structures of TCPP (**Figure S3**). Matrix-assisted laser desorption/ionization time-of-flight mass spectrometry (MALDI-TOF-MS) showed that the molecular mass of TCPP and Mn-TCPP was 791.34 and 845.22, respectively, consistent with their theoretical values. MOF and Mn-MOF was then synthesized by solvothermal reaction between zirconyl chloride octahydrate (ZrOCl_2_·8H_2_O) and TCPP or Mn-TCPP in *N*,*N*-Dimethylformamide (DMF) at 90 °C for 5 h. The resulting MOF (the conventional PCN-224 with a monomer molecular formula of Zr_6_C_72_H_45_N_6_O_12_) and Mn-MOF (the monomer molecular formula of Zr_6_C_72_H_45_N_6_O_12_Mn_1.5_) were washed with DMF and deionized water and then suspended in deionized water for further experiments. The loading efficiency of Mn-TCPP in Mn-MOF was 69.67%.

**Figure 1:**
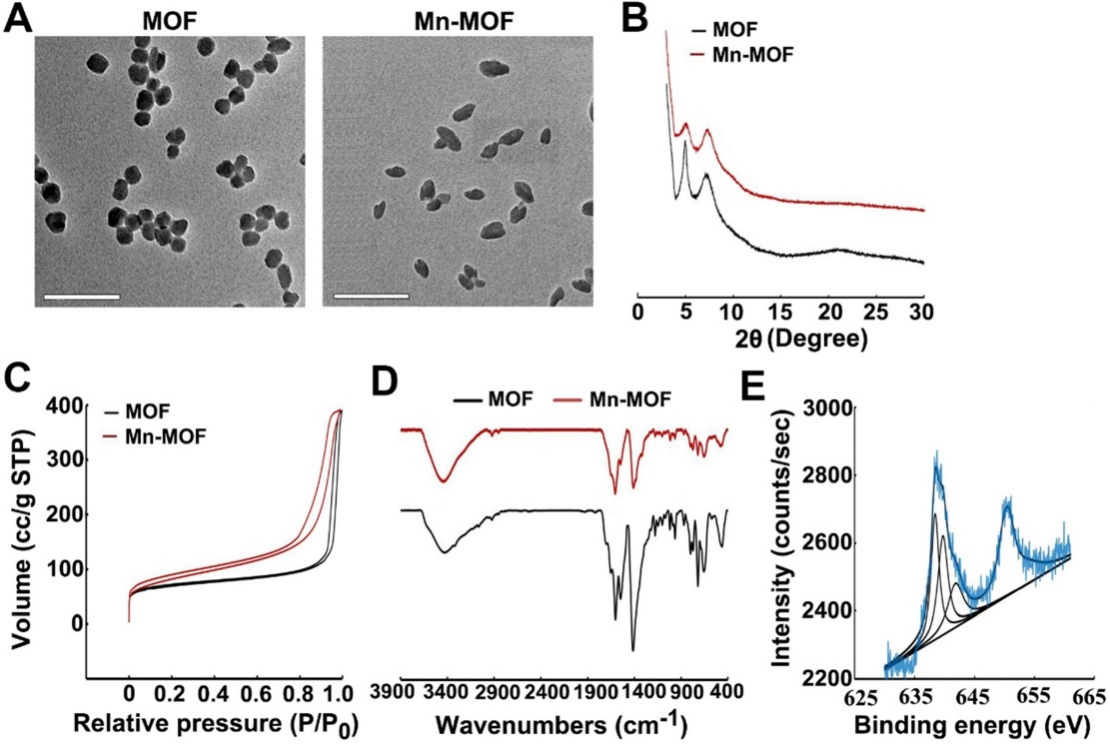
Characterization of Mn-MOF. (A) TEM images of MOF and Mn-MOF. Scale bar: 200 nm. (B) PXRD patterns of MOF and Mn-MOF. (C) N_2_ adsorption/desorption isotherms of MOF and Mn-MOF. (D) FT-IR spectra of MOF and Mn-MOF. (E) Valence analysis of Mn in Mn-MOF.

Dynamic light scattering (DLS) analysis revealed that Mn-MOF and MOF exhibited similar diameter of 70.0 nm and 68.9 nm (**Figure S4A**), and zeta potential of 24.3 mV and 25.1 mV (**Figure S4B**), respectively. Transmission electron microscopy (TEM) images showed spherical-like morphology for Mn-MOF and MOF (**Figure 1A**). Powder X-ray diffraction (PXRD) indicated that the as-synthesized Mn-MOF and MOF were highly crystalline (**Figure 1B**). The distance between secondary building units (SBUs) was measured to be about 1.2 nm by high-resolution TEM (**Figure S5**). The BET surface area, pore volume and average pore diameter as measured by nitrogen adsorption analysis were 292.1 m^2^/g, 0.605 cm^3^/g and 1.2 nm for Mn-MOF, respectively, and 250.6 m^2^/g, 0.602 cm^3^/g and 1.1 nm for MOF, respectively (**Figure 1C**). Moreover, the diameter of Mn-MOF and MOF remained almost constant after incubation in RPMI 1640 medium for 7 days, suggesting that Mn-MOF and MOF are relatively stable (**Figure S6**).

UV-visible absorption spectroscopy indicated that TCPP has a major Soret peak at λ_max_ = 416 nm and four Q-bands at 513, 547, 590 and 645 nm. In comparison, the Soret peak of Mn-TCPP is at λ_max_ = 465 nm and the Q-bands are at 564 nm and 597 nm (**Figure S7A**). Mn-MOF and MOF exhibited the similar UV-visible absorption pattern to Mn-TCPP and TCPP, respectively (**Figure S7B**), suggesting that Mn-TCPP and TCPP were successfully integrated into Mn-MOF and MOF, respectively, and metal node Zr did not significantly affect the UV-vis absorption spectra of organic linkers. FT-IR analysis also confirmed that Mn-MOF and MOF were successfully synthesized (**Figure 1D**). Energy-dispersive X-ray spectroscopy (EDX) elemental mapping showed a uniform distribution of C, N, O, Zr and Mn in Mn-MOF, while only C, N, O and Zr were dispersed in MOF (**Figure S8**). The X-ray photoelectron spectroscopy (XPS) spectra confirmed the presence of these elements in Mn-MOF and MOF (**Figure S9**). The peak at 636 eV and 661 eV in wide-scan XPS spectra of Mn-TCPP and Mn-MOF indicated the presence of Mn 2p3/2, which can be deconvoluted into three components, Mn^2+^ (638.5 eV), Mn^3+^(639.7 eV) and Mn^4+^ (641.5 eV) (**Figure 1E** and **Figure S10**). Inductively coupled plasma-atomic emission spectrometry (ICP-AES) analysis showed that the Zr/Mn atom ratio was 3 in Mn-MOF. The difference between theoretical value and actual measurement value of Zr/Mn ratio might be that crystal defects occurred randomly during the preparation of Mn-MOF, affecting the coordination saturation. In addition, the addition of benzoic acid caused the simultaneous loss of Zr clusters and organic ligands, resulting in the deviation of Zr/Mn ratio.

### Catalase-like and GSH decreasing activity of Mn-MOF for SDT* in vitro*

**Figure 2:**
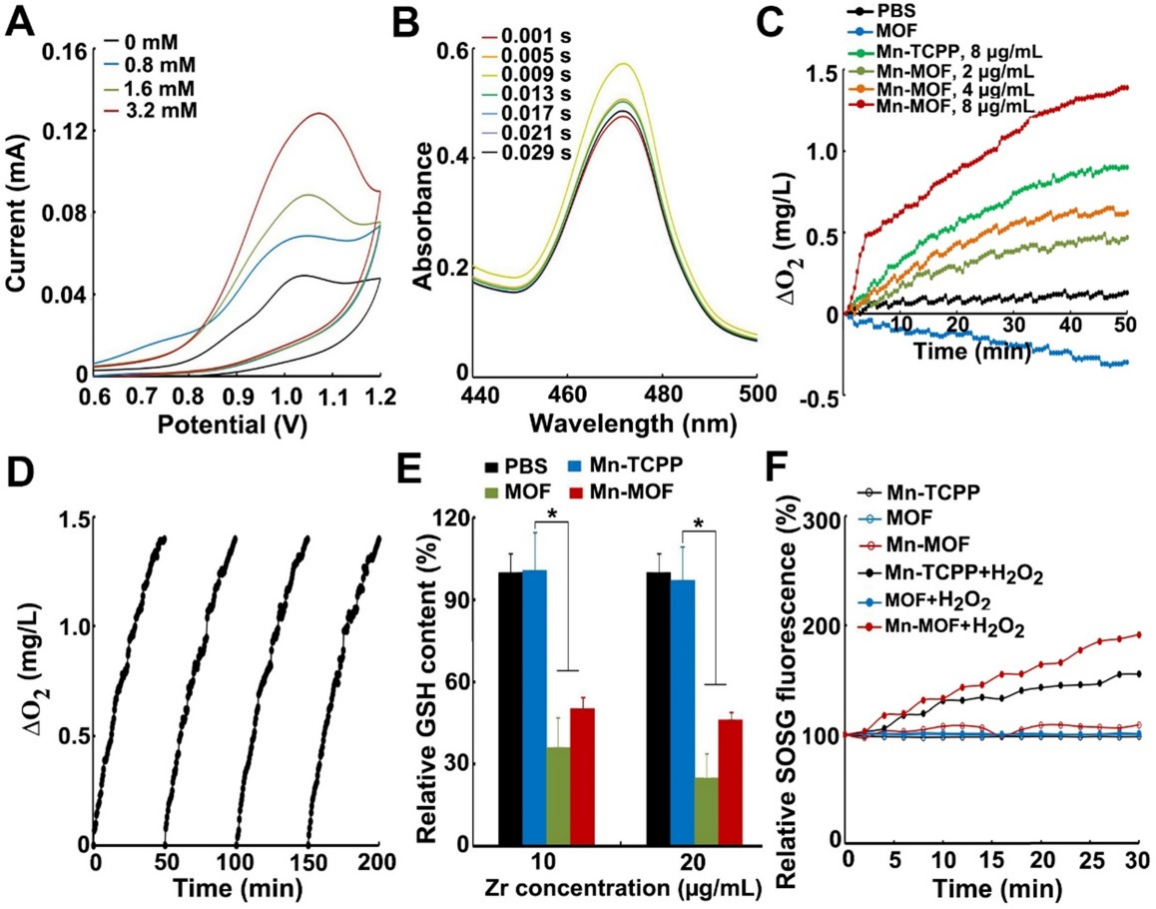
*In vitro* catalase-like and GSH decreasing activity of Mn-MOF for SDT treatment. (A) Cyclic voltammetry of Mn-MOF modified electrode at Mn concentration of 8 µg/mL in different concentrations of H_2_O_2_. (B) Stopped-flow spectra of the reaction kinetics between Mn-MOF at the Mn concentration of 8 µg/mL and 400 µM H_2_O_2_. (C) O_2_ generation after Mn-TCPP, MOF and Mn-MOF at different Mn concentrations (the concentration of MOF was quantified according to the Zr concentration of Mn-MOF at Mn concentration of 8 µg/mL) were treated with 400 µM H_2_O_2_ for different time intervals. (D) Persistent O_2_ generation of Mn-MOF at Mn concentration of 8 µg/mL subjected to four cycles of 400 µM H_2_O_2_ treatment. (E) GSH consumption after Mn-TCPP, MOF and Mn-MOF at the Zr concentration of 10 and 20 µg/mL (the corresponding Mn concentration was 2 and 4 µg/mL for Mn-TCPP, respectively) were incubated with 5 µM GSH for 40 min. (F) ROS generation after Mn-TCPP, MOF and Mn-MOF at the Zr concentration of 50 µg/mL and Mn concentration of 10 µg/mL were treated with or without 400 µM H_2_O_2_ for different time intervals under hypoxic conditions upon US irradiation (1 MHz, 0.9 W/cm^2^, 30% duty cycle). The data are presented as mean ± s.d. (n = 3). *P < 0.05.

Manganese porphyrins undergo reversible one-electron oxidation and reduction under physiological conditions, exhibiting catalase-like function in which Mn^3+^-porphyrins and Mn^4+^-porphyrins experience cyclic conversion upon H_2_O_2_ addition 20. To demonstrate whether Mn-MOF possessed similar activity, the electrochemical behaviors of the Mn-MOF-modified glass carbon electrode (GCE) in H_2_O_2_ at different concentrations were evaluated through cyclic voltammetry (CV) (**Figure 2A**). The strong characteristic oxidation peak at 1.05 V in cyclic voltammograms was dominated by the Mn^3+^/Mn^4+^ redox couple of Mn-MOF 30. The currents of Mn-MOF-modified GCE at 1.05 V increased in a H_2_O_2_ concentration-dependent manner, suggesting that Mn^3+^ was oxidized to Mn^4+^ in Mn-MOF after reaction with H_2_O_2_. Furthermore, stopped-flow spectroscopy was used to analyze the dynamic change of absorbance in Mn-MOF after reaction with H_2_O_2_ (**Figure 2B**). The absorbance at 471 nm, a typical Mn^3+^-porphyrins absorption peak 31, increased quickly upon H_2_O_2_ addition, then decreased and tended to balance over time, which might be due to the dynamic conversion of Mn^3+^/Mn^4+^ in Mn-MOF after reaction with H_2_O_2_. These results suggested that Mn-MOF might exhibit catalase-like activity.

H_2_O_2_ could be decomposed by catalase to generate O_2_. To further clarify the catalase-like activity of Mn-MOF, the decomposition of H_2_O_2_ was first detected by a H_2_O_2_ kit. About 68.7% of H_2_O_2_ was decomposed after treatment with Mn-MOF for 8 h, higher than Mn-TCPP group (**Figure S11**), revealing the efficient catalytic activity of Mn-MOF. In contrast, MOF did not show obvious catalysis of H_2_O_2_ (**Figure S11**). Correspondingly, Mn-MOF exhibited a concentration-dependent increase in O_2_ generation after addition of H_2_O_2_ as measured by a dissolved oxygen meter (**Figure 2C**). The Mn-MOF-induced O_2_ generation upon H_2_O_2_ addition was stronger than Mn-TCPP group (**Figure 2C**), which might be that the large surface area of Mn-MOF was easier to react with H_2_O_2_. Conversely, MOF did not enhance O_2_ production in response to H_2_O_2_ (**Figure 2C**). The ability of Mn-MOF to catalyze H_2_O_2_ to produce O_2_ was further confirmed by the fact that the addition of more H_2_O_2_ can generate more O_2_ (**Figure S12**). More importantly, Mn-MOF could persistently catalyze H_2_O_2_ to generate O_2_, as evidenced by the fact that Mn-MOF still remained good catalytic activity of H_2_O_2_ even undergoing four cycles (**Figure 2D**).

Recent works showed that redox-active metal ions such as copper(II) tend to absorb and oxidize GSH, decreasing the intracellular GSH level 32,33. In addition to O_2_ supplementation, Mn-MOF could efficiently decrease GSH concentration. About 80% of GSH was downregulated by Mn-MOF and MOF after incubation for 40 min as measured by a GSH kit, while Mn-TCPP did not affect GSH content (**Figure 2E**), suggesting that Zr in Mn-MOF and MOF could decrease GSH. The Mn-MOF- and MOF-induced GSH decrease was further confirmed after incubation with higher concentration of GSH for longer time (**Figure S13**).

Since SDT effect is highly dependent on O_2_ concentration, the ROS generation induced by Mn-MOF upon US irradiation in the presence or absence of H_2_O_2_ under normoxic or hypoxic conditions was determined by singlet oxygen sensor green (SOSG). Under normoxic conditions, Mn-TCPP, MOF and Mn-MOF could efficiently generate ROS after US irradiation (**Figure S14**). However, under hypoxia, MOF did not generate ROS in the presence or absence of H_2_O_2_ after US irradiation (**Figure 2F**). In contrast, US irradiation treatment significantly increased ROS production induced by Mn-MOF after addition of H_2_O_2,_ even under hypoxic condition (**Figure 2F** and **Figure S14**). Importantly, the Mn-MOF-induced ROS generation in the presence of H_2_O_2_ upon US irradiation was stronger than Mn-TCPP-treated group (**Figure 2F** and**Figure S14**), which might be due to more O_2_ generation induced by Mn-MOF. These results further confirmed that Mn-MOF, with the assistance of catalase-like and GSH decreasing activity, could efficiently produce O_2_ in response to H_2_O_2_ and reduce GSH, generating good SDT effect under hypoxia.

### *In vitro* SDT and ferroptosis effects of Mn-MOF

**Figure 3:**
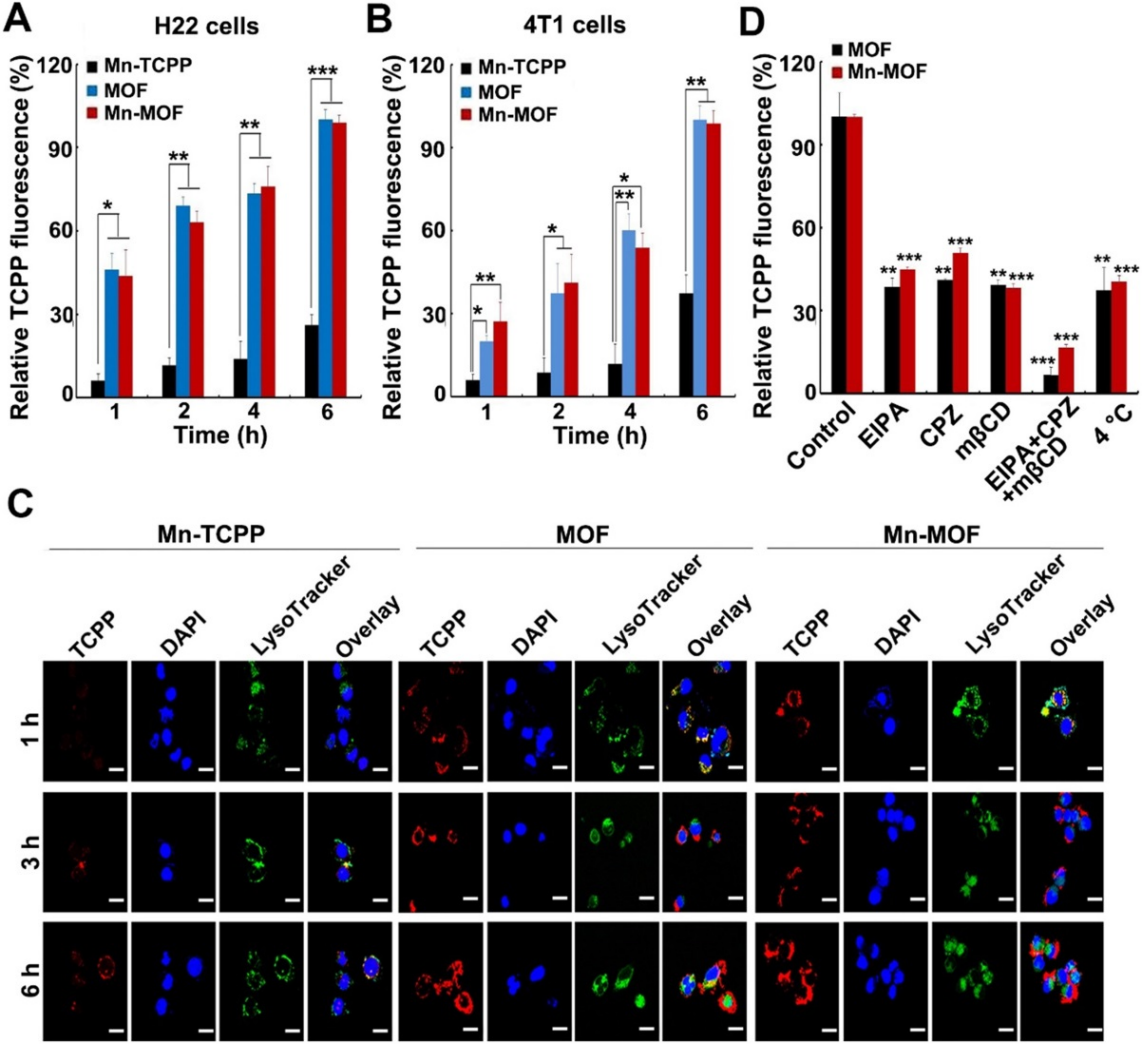
Cellular uptake and intracellular location of Mn-MOF. (A,B) Relative TCPP fluorescence intensity in H22 (A) and 4T1 cells (B) treated with Mn-TCPP, MOF or Mn-MOF at the Zr concentration of 10 µg/mL and Mn concentration of 2 µg/mL for different time intervals. (C) Confocal microscopic images of 4T1 cells treated with Mn-TCPP, MOF or Mn-MOF at the Zr concentration of 10 µg/mL and Mn concentration of 2 µg/mL for different time intervals, and then labeled with 5 μg/mL DAPI and 50 nM LysoTracker green. Scale bar: 20 µM. (D) Relative TCPP fluorescence intensity in 4T1 cells treated with the specific endocytic inhibitors as above, followed by treatment with Mn-TCPP, MOF or Mn-MOF at the Zr concentration of 10 µg/mL and Mn concentration of 2 µg/mL for 2 h. The data are presented as mean ± s.d. (n = 3). *P < 0.05, **P < 0.01, ***P < 0.001.

In order to evaluate the SDT and ferroptosis effects of Mn-MOF, the cellular uptake of Mn-MOF was first determined in H22 and 4T1 cells by flow cytometry and confocal microscopy (**Figure 3A-C**). The cellular accumulation of Mn-TCPP, MOF and Mn-MOF increased in a time-dependent manner. However, the intracellular accumulation of Mn-MOF and MOF in these cells was significantly higher than that of Mn-TCPP. Meanwhile, Mn-MOF, MOF and Mn-TCPP were mainly located in lysosomes after internalization (**Figure 3C**). 5-(*N*-ethyl-*N*-isopropyl) (EIPA, an inhibitor of macropinocytosis), chlorpromazine (CPZ, an inhibitor of clathrin-mediated endocytosis) or methyl-*b*-cyclodextrin (mβCD, an inhibitor of caveolae-dependent endocytosis) markedly reduced the cellular uptake of MOF and Mn-MOF (**Figure 3D**). Combination of EIPA, CPZ and mβCD further decreased the cellular uptake of MOF and Mn-MOF (**Figure 3D**), suggesting that clathrin, caveolae and macropinocytosis were involved in the endocytosis of MOF and Mn-MOF.

**Figure 4:**
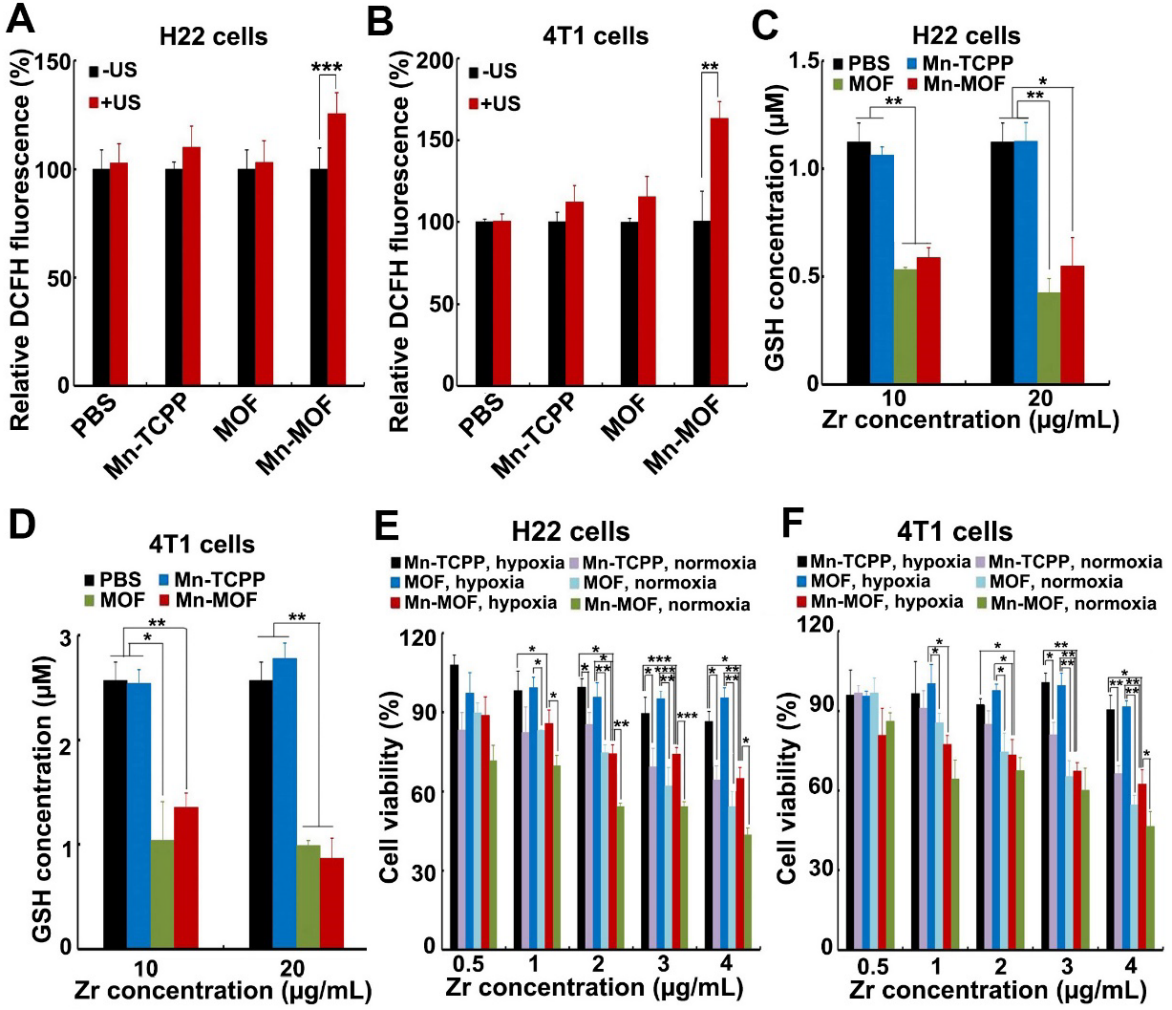
*In vitro* SDT effects induced by Mn-MOF. (A,B) ROS generation in H22 (A) and 4T1 cells (B) treated with Mn-TCPP, MOF or Mn-MOF at the Zr concentration of 10 µg/mL and Mn concentration of 2 µg/mL for 10 h under hypoxia in the presence or absence of US irradiation (1 MHz, 0.9 W/cm^2^, 30% duty cycle). (C,D) Intracellular GSH content in H22 (C) and 4T1 (D) cells after treatment with Mn-TCPP, MOF or Mn-MOF at the Zr concentration of 10 and 20 µg/mL (the corresponding Mn concentration was 2 and 4 µg/mL) for 24 h. (E,F) Cell viability of H22 (E) and 4T1 cells (F) after treatment with Mn-TCPP, MOF or Mn-MOF at the different Zr concentrations (the quantification of Mn-TCPP was calculated according to the corresponding Mn concentration of Mn-MOF) for 10 h under normoxia or hypoxia, followed by US irradiation (1 MHz, 0.9 W/cm^2^, 30% duty cycle) for 10 min. The data are presented as mean ± s.d. (n = 3). *P < 0.05, **P < 0.01, ***P < 0.001.

The ability of Mn-MOF to trigger ROS generation upon US irradiation under normoxic and hypoxic conditions was further examined by using 2',7'-dichlorofluorescein diacetate (DCFH-DA) that could be rapidly oxidized by ROS to emit fluorescence 34,35. As expected, under normoxia, stronger green fluorescence was detected in both MOF- and Mn-MOF-treated H22 and 4T1 cells upon US irradiation compared with those without US irradiation group (**Figure S15** and**Figure S16**). In contrast, no significant fluorescence increase was detected in Mn-TCPP- and MOF-treated groups under hypoxia after US irradiation, while US irradiation significantly enhanced the green fluorescence intensity induced by Mn-MOF under hypoxic condition (**Figure 4A-B** and**Figure S16**), suggesting that Mn-MOF produced amounts of ROS upon US irradiation under hypoxia. Such high ROS generation induced by Mn-MOF might be due to the sufficient O_2_ supplementation via catalyzing endogenous H_2_O_2_ and down-regulation of GSH to protect the generated ROS. Indeed, Mn-MOF significantly decreased intracellular GSH content in H22 and 4T1 cells (**Figure 4C-D**). Next, we evaluated the cytotoxicity of Mn-MOF against H22 and 4T1 cells under normoxic and hypoxic conditions upon US irradiation. Mn-TCPP, MOF and Mn-MOF did not significantly affect the cell viability of H22 and 4T1 cells under both hypoxia and normoxia (**Figure S17**), revealing good biocompatibility. Under normoxia, both MOF and Mn-MOF could efficiently kill cancer cells upon US irradiation (**Figure 4E-F**). In contrast, under hypoxia, MOF did not significantly inhibit cancer cell viability upon US irradiation, while Mn-MOF exhibited the dose-dependent cytotoxicity upon US irradiation (**Figure 4E-F**). IC_50_ value of Mn-MOF confirmed its good cytotoxicity against cancer cells under hypoxia (**Table S1**). These results suggested that Mn-MOF exhibited strong cytotoxicity against cancer cells upon US irradiation under hypoxia due to good SDT effects. Mn-TCPP exhibited low cytotoxicity under both hypoxia and normoxia (**Figure 4E-F**), which might be related with the low internalization into cancer cells.

**Figure 5:**
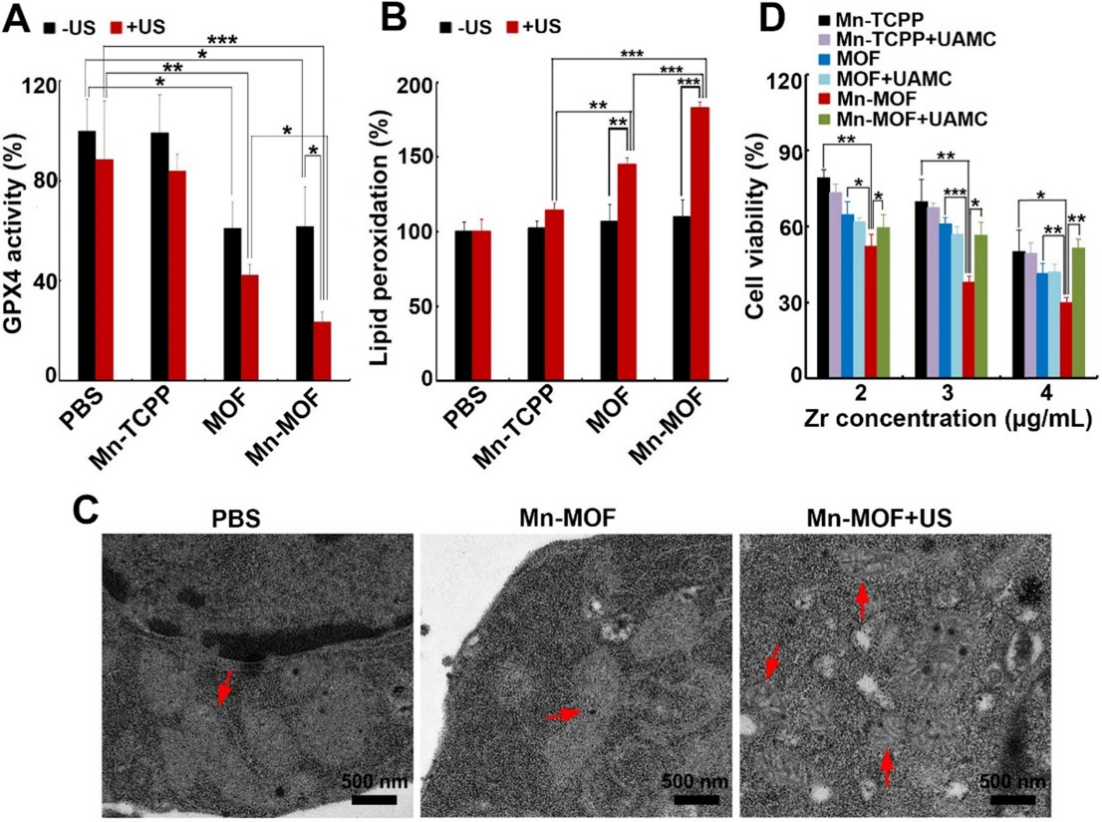
Mn-MOF-induced ferroptosis upon US irradiation. (A) GPX4 activity of H22 cells treated with Mn-TCPP, MOF or Mn-MOF at the Zr concentration of 10 µg/mL and Mn concentration of 2 µg/mL for 10 h in the presence or absence of US irradiation (1 MHz, 0.9 W/cm^2^, 30% duty cycle). (B) LPO levels of H22 cells treated with Mn-TCPP, MOF or Mn-MOF at the Zr concentration of 10 µg/mL and Mn concentration of 2 µg/mL for 10 h in the presence or absence of US irradiation (1 MHz, 0.9 W/cm^2^, 30% duty cycle). (C) TEM images of H22 cells after treatment with Mn-MOF at the Zr concentration of 10 µg/mL for 10 h in the presence or absence of US irradiation (1 MHz, 0.9 W/cm^2^, 30% duty cycle). (D) Cell viability of H22 cells treated with Mn-TCPP, MOF or Mn-MOF at the different Zr concentrations (the quantification of Mn-TCPP was calculated according to the corresponding Mn concentration of Mn-MOF) in the presence or absence of 10 nM UAMC-3203 for 10 h under normoxia for 10 h, followed by US irradiation (1 MHz, 0.9 W/cm^2^, 30% duty cycle) for 10 min. The data are presented as mean ± s.d. (n = 3). *P < 0.05, **P < 0.01, ***P < 0.001.

GPX4 is a central regulator of ferroptosis that combats with LPO and prevents ferroptotic cell death, whose activity is controlled by GSH content 10. In view that Mn-MOF efficiently depleted GSH, and ZrO_2_ nanoparticles were reported to decrease GPX activity 36, GPX4 activity was evaluated in H22 cells treated with Mn-TCPP, MOF or Mn-MOF in the presence or absence of US irradiation. As expected, Mn-MOF and MOF significantly decreased GPX4 activity, and US irradiation further decreased Mn-MOF-induced GPX4 inhibition (**Figure 5A**). The accumulation of LPO is considered as a hallmark of ferroptosis 7-9. ROS can cause cancer cell death by damaging biomolecules like DNA/RNA, proteins and lipids. To determine whether Mn-MOF induced LPO upon US irradiation, H22 cells were treated with Mn-TCPP, MOF or Mn-MOF, followed by US irradiation. Consistently, Mn-MOF exhibited the strongest LPO levels upon US irradiation compared with other groups (**Figure 5B**), suggesting that Mn-MOF might induce ferroptosis upon US irradiation. Furthermore, the TEM results showed that the mitochondria appeared smaller in Mn-MOF-treated H22 cells upon US irradiation than Mn-MOF without US irradiation- and PBS-treated groups (**Figure 5C**), exhibiting the typical morphological changes of ferroptosis. To determine whether Mn-MOF-induced ferroptosis upon US irradiation was responsible for the cell cytotoxicity, H22 cells were treated with Mn-MOF upon US irradiation in ther presence or absence of the inhibitor of ferroptosis, UAMC-3203 or Ferrostatin-1 (Fer-1), and the cell cytotoxicity was then assessed. The results showed that Mn-MOF-induced cytotoxicity upon US irradiation was efficiently abrogated after treatment with UAMC-3203 (**Figure 5D and Figure S18**) or Fer-1 (**Figure S19**) under normoxia and hypoxia. These results suggested that Mn-MOF displayed excellent anticancer effects upon US irradiation under hypoxia owing to the SDT effects and ferroptosis.

### *In vivo* anticancer activity of Mn-MOF upon US irradiation in H22 tumor-bearing mice

To achieve systemic administration, Mn-MOF and MOF were further modified with BSA. BSA modificaition increased the size of Mn-MOF and MOF (**Figure S20A**), and shifted their zeta potential to negative charged (**Figure S20B**). Meanwhile, BSA modification did not significantly change the Mn-MOF-induced H_2_O_2_ decompostion (**Figure S20C**), O_2_ generation in response to H_2_O_2_ (**Figure S20D**), and ROS generation upon US irradiation in the presence of H_2_O_2_ under hypoxic condition (**Figure S20E**), suggesting that BSA modification did not affect the function of Mn-MOF (BSA-modified Mn-MOF and MOF in the following* in vivo* experiments were still referred as Mn-MOF and MOF). To evaluate the tumor targeting capacity of Mn-MOF via enhanced permeability and retention (EPR) effect, H22 tumor-bearing mice were intravenously injected with Mn-TCPP, MOF or Mn-MOF. At 24 h after administration, the tumors and major organs (heart, liver, spleen, lung and kidney) of the mice were collected, and Mn and Zr content in these tissues were determined by inductively coupled plasma-mass spectrometry (ICP-MS). Mn-MOF, similar to MOF, exhibited strong tumor accumulation, about 2.0 times relative to Mn-TCPP (**Figure S21**), suggesting the efficient passive tumor homing of Mn-MOF.

**Figure 6:**
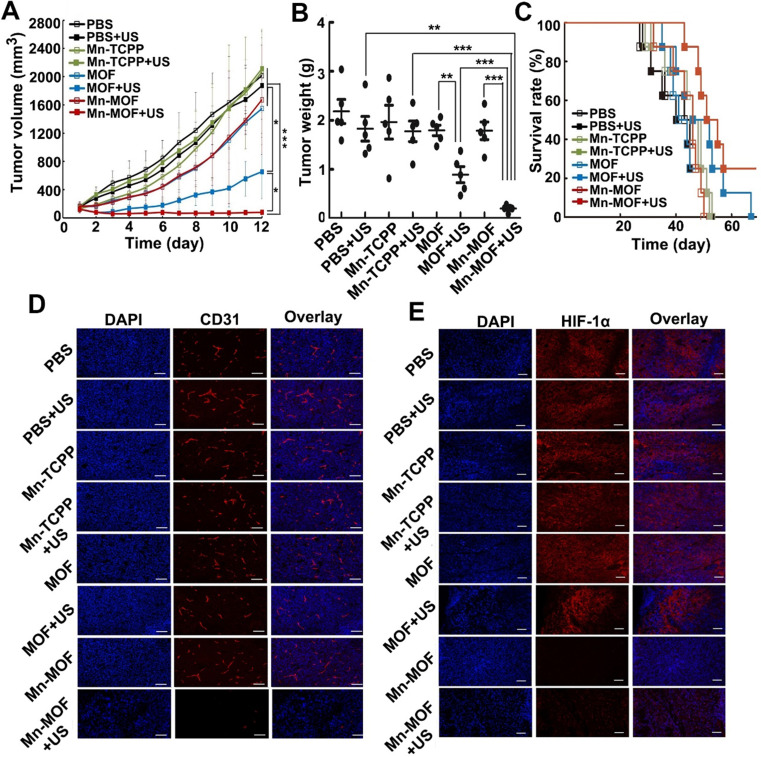
*In vivo* SDT effects of Mn-MOF in H22 tumor-bearing mice. (A) Tumor growth curves of H22 tumor-bearing mice after intravenous injection of PBS, Mn-TCPP, MOF or Mn-MOF at the Zr dosage of 5 mg/kg and Mn dosage of 1 mg/kg, followed by US irradiation (1 MHz, 1.0 W/cm^2^, 50% duty cycle) for 10 min. The data are presented as mean ± s.d. (n= 13). (B) Tumor weight at the end of treatment indicated in A. The data are presented as mean ± s.d. (n= 5). *P < 0.05, **P < 0.01, ***P < 0.001. (C) Kaplan-Meier survival plot of H22 tumor-bearing mice after treatment indicated in A (n= 8). (D,E) Immunofluorescent staining of CD31-labeled vessels (D) and HIF-1α (E) in tumor tissues of mice after treatment indicated in A. Scale bar: 50 µm.

The* in vivo* anticancer activity of Mn-MOF upon US irradiation were then evaluated in H22 tumor-bearing mice. The mice were intravenously injected with Mn-TCPP, MOF or Mn-MOF, followed with or without US irradiation at 24 h after injection. The tumors grew rapidly in the PBS-treated group. Mn-TCPP, MOF and Mn-MOF did not significantly inhibit the tumor growth. Upon US irradiation, although Mn-TCPP did not markedly affect the tumor growth due to the insufficient tumor targeting, MOF significantly halted the development and progression of the tumor mass with 51.4% tumor inhibition (**Figure 6A**). However, Mn-MOF exhibited the strongest inhibitory effect, achieving 89.2% tumor shrinkage (**Figure 6A**). The average weight of the tumor tissues excised at the end of treatment also exhibited the same trend (**Figure 6B**). Kaplan-Meier survival analysis showed that 25% of mice remained alive in Mn-MOF-treated group upon US irradiation after 68 days when all mice in other groups had died (**Figure 6C**). Hematoxylin and eosin (H&E) staining revealed that prominently enhanced tumor necrosis was observed in the tumor slices of the mice treated with Mn-MOF upon US irradiation (**Figure S22**). The excellent anticancer activity of Mn-MOF upon US irradiation was effeicently abrogated after treatment with UAMC-3203 (**Figure S23**), suggesting that ferroptosis was responsible for the anticancer activity of Mn-MOF upon US irradiation. These results showed that the excellent anticancer activity of Mn-MOF upon US irradiation was related to the synergistic activity of SDT effects and ferroptosis.

Besides directly killing tumor cells, ROS can induce microvascular collapse 37. To further evaluate the Mn-MOF-induced anticancer effects upon US irradiation, CD31-labeled tumor vessels were examined after treatment (**Figure 6D**). Compared with other groups, Mn-MOF significantly destroyed tumor vessels upon US irradiation, which might inhibit tumor growth. The vascular collapse is unfavourable to the O_2_ supply and aggravates the hypoxia microenvironment 38. To determine the ability of Mn-MOF to ameliorate tumor hypoxia, hypoxia-inducible factor (HIF)-1α as an indicator of tumor hypoxia was evaluated since the hypoxic condition within the tumor microenvironment can induce the expression of HIF-1α 39 (**Figure 6E**). Compared with other groups, the tumors of mice treated with Mn-MOF in the presence or absence of US irradiation showed very weak red fluorescence from HIF-1α staining, confirming that the tumor hypoxia was greatly alleviated by Mn-MOF through the decomposition of tumor endogenous H_2_O_2_ to *in-situ* generate O_2_.

*In vivo* side effects of Mn-MOF were comprehensively conducted by monitoring body weight during treatment, serological analysis and H&E staining of major organs after treatment. No significant change in body weight was observed in Mn-MOF-treated group during treatment (**Figure S24**). All blood indexes in Mn-MOF-treated group, including alanine aminotransferase (ALT), aspartate aminotransferase (AST), blood urea nitrogen (BUN), creatine kinase (CK), red blood cell count (RBC), white blood cell count (WBC), platelet count and hemoglobin did not show significant differences as compared with the control group (**Figure S25**). Meanwhile, no obvious pathological changes were detected in the H&E staining of main organs in each group (**Figure S22**). These results indicated that Mn-MOF exhibited good biocompatibility.

### *In vivo* anticancer activity of Mn-MOF upon US irradiation in metastatic 4T1 tumor-bearing mice

**Figure 7:**
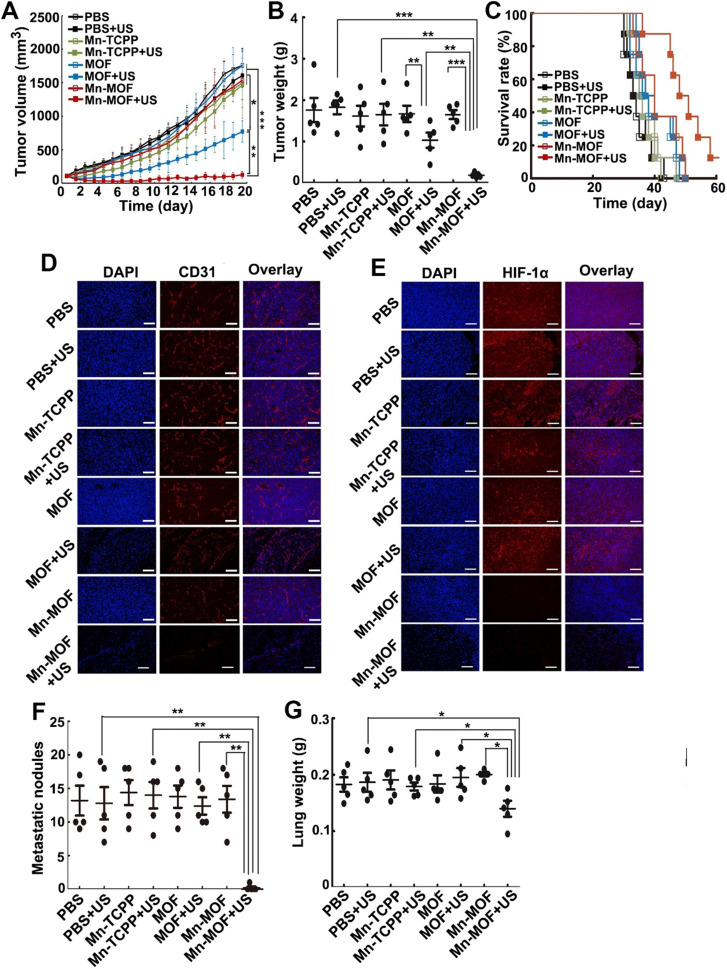
* In vivo* SDT effects of Mn-MOF in metastatic 4T1 tumor-bearing mice. (A) Tumor growth curves of 4T1 tumor-bearing mice after intravenous injection of PBS, Mn-TCPP, MOF or Mn-MOF at the Zr dosage of 5 mg/kg and Mn dosage of 1 mg/kg, followed by US irradiation (1 MHz, 1.0 W/cm^2^, 50% duty cycle) for 5 min. The data are presented as mean ± s.d. (n= 13). (B) Tumor weight at the end of treatment indicated in A. The data are presented as mean ± s.d. (n= 5). (C) Kaplan-Meier survival plot of 4T1 tumor-bearing mice after treatment indicated in A (n= 8). (D,E) Immunofluorescent staining of CD31-labeled vessels (D) and HIF-1α (E) in tumor tissues of mice after treatment indicated in A. Scale bar: 50 μm. (F,G) Pulmonary metastatic nodules (F) and lung weight (G) in 4T1 tumor-bearing mice after treatment indicated in A. The data are presented as mean ± s.d. (n= 5). *P < 0.05, **P < 0.01, ***P < 0.001.

To further investigate the anticancer effects of Mn-MOF upon US irradiation, the mice bearing subcutaneous 4T1 metastatic breast tumors were intravenously injected with Mn-TCPP, MOF or Mn-MOF, followed with or without US irradiation at 24 h after injection. As expected, Mn-MOF exhibited the strongest anticancer activity upon US irradiation, with 93.4% and 90.8% reduction in tumor volume and tumor weight compared with PBS group, respectively (**Figure 7A-B**). Still 12.5% of mice remained alive in Mn-MOF-treated group upon US irradiation after 60 days, although all mice in other group died before 51 days (**Figure 7C**). These results further confirmed the excellent anticacer activity of Mn-MOF upon US irradiation. Consistently, Mn-MOF dramatically destroyed the tumor vessels upon US irradiation (**Figure 7D**) and the tumor hypoxia was significantly improved by Mn-MOF, as evidenced by the fact that HIF-1α expression was lower in the tumors of Mn-MOF-treated mice (**Figure 7E**), thereby down-regulating the expression of vascular endothelial growth factor (VEGF) (**Figure S26**).

Tumor hypoxia not only induces the severe resistance to SDT but also promotes tumor metastasis and progression 40,41. Thus, the antimetastatic efficacy of Mn-MOF upon US irradiation was then evaluated. Significantly fewer metastatic nodules (**Figure 7F**) and decreased lung weight (**Figure 7G**) were detected in Mn-MOF-treated group upon US irradiation. The inhibitory rate of metastatic nodules of Mn-MOF-treated group upon US irradiation was 96.9% compared with PBS group. No significant inhibition in lung metastasis was observed in Mn-TCPP- or MOF-treated mice upon US irradiation (**Figure 7F-G**). The less lung metastasis in Mn-MOF-treated mice upon US irradiation was further confirmed by H&E staining on lungs (**Figure S27**). Besides lung, no obvious pathological changes were detected in the H&E staining of other main organs, including heart, liver, spleen and kidney in each group (**Figure S27**). These results showed that Mn-MOF exhibited the excellent antimetastatic activity and good biocompatibility upon US irradiation.

### Mn-MOF-induced antitumor immunity upon US irradiation

**Figure 8:**
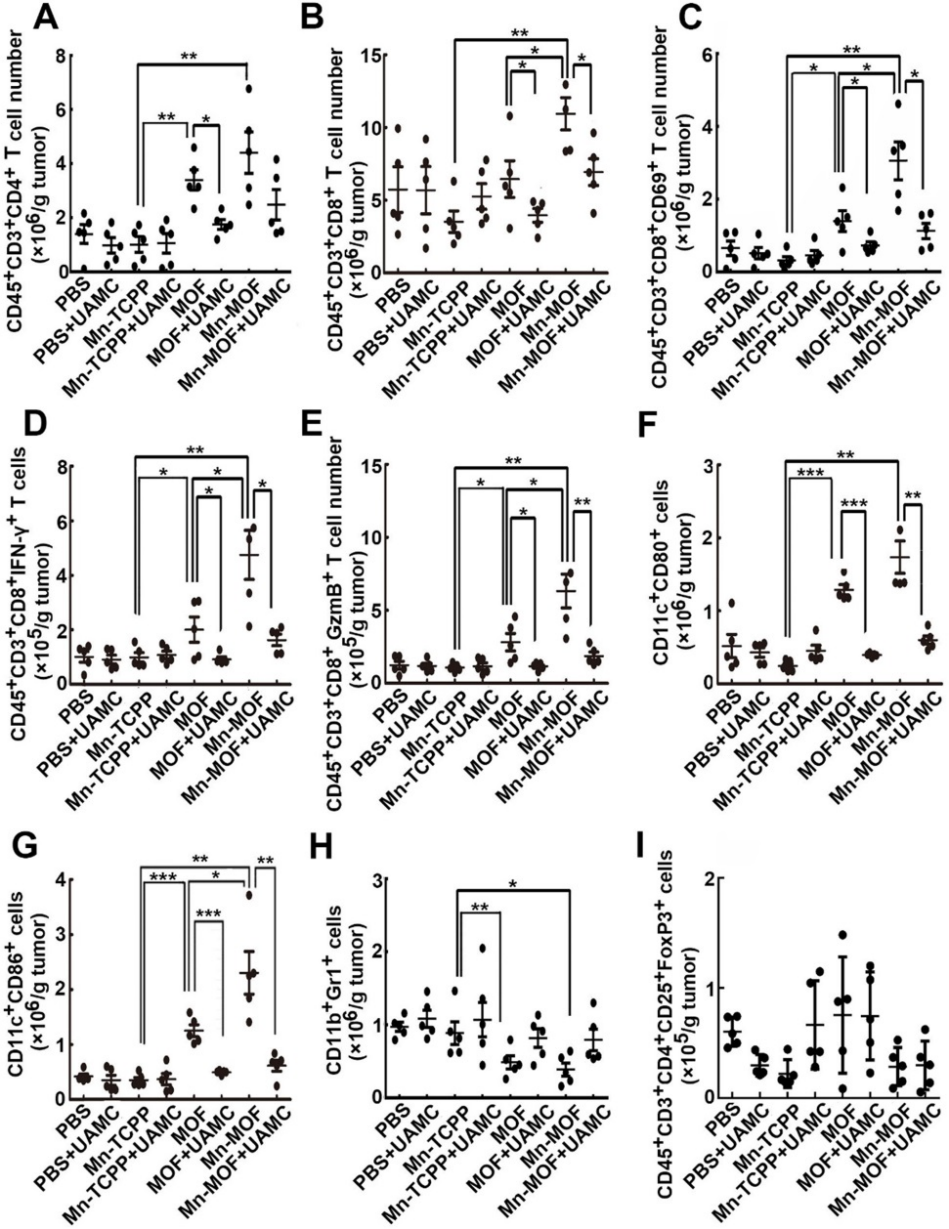
Involvement of ferroptosis in the Mn-MOF-induced improved tumor immunity. (A-I) The numbers of CD4^+^ T cells (A), CD8^+^ T cells (B), CD8^+^CD69^+^ T cells (C), CD8^+^IFN-γ^+^ T cells (D), CD8^+^GzmB^+^ T cells (E), CD11c^+^CD80^+^ dendritic cells (F), CD11c^+^CD86^+^ dendritic cells (G), MDSCs (H) and Tregs (I) in tumor tissues of H22 tumor-bearing mice after intravenous injection of PBS, Mn-TCPP, MOF or Mn-MOF at Zr dosage of 5 mg/kg (the corresponding Mn dosage was 1 mg/kg) in the presence or absence of intraperitoneal injection of UAMC-3203 at 20 μg/kg dosage, followed by US irradiation (1.0 MHz, 1 W/cm^2^, 50% duty cycle) at tumors for 10 min. The data are presented as mean ± s.d. (n= 5). *P < 0.05, **P < 0.01, ***P < 0.001.

ROS efficiently activates the antitumor immunity 42, and ferroptosis was also reported to be involved in T cell-mediated antitumor immunity 43. To determine whether Mn-MOF affected the tumor immune microenvironment upon US irradiation and ferroptosis was involved in Mn-MOF-induced antitumor immunity, the numbers of immune cells in tumor tissues were determined in H22 tumor-bearing mice after intravenous injection of PBS, Mn-TCPP, MOF or Mn-MOF in the presence or absence of intraperitoneal injection of UAMC-3203, followd by US irradiation at 24 h after injection. Compared with Mn-TCPP and MOF, Mn-MOF exhibited the strongest capacity to enhance the numbers of CD4^+^ T cells (**Figure 8A**) and CD8^+^ T cells (**Figure 8B**) upon US irradiation. Meanwhile, the numbers of CD8^+^CD69^+^ T cells (**Figure 8C**), CD8^+^IFN-γ^+^ T cells (**Figure 8D**) and CD8^+^Granzyme B^+^ (GzmB^+^) T cells (**Figure 8E**) were significantly enhanced in Mn-MOF-treated group upon US irradiation, suggesting that Mn-MOF efficiently activated CD8^+^ T cells upon US irradiation. Mn-MOF significantly increased the numbers of matured CD11c^+^CD80^+^ (**Figure 8F**) and CD11c^+^CD86^+^ (**Figure 8G**) dendritic cells (DCs), while significantly decreased the numbers of MDSCs (**Figure 8H**), not regulatory T cells (Tregs) (**Figure 8I**) in tumor tissues upon US irradiation, these results suggested that Mn-MOF efficiently activated the antitumor immunity and ameliorated the tumor immunosuppression upon US irradiation. However, UAMC-3203 significantly decreased the numbers of Mn-MOF-enhanced CD8^+^ T cells, activated CD8^+^ T cells and matured DCs upon US irradiation in tumor tissues (**Figure 8A-I**), revealing that ferroptosis might be responsible for the improved tumor immune microenvironment provoked by Mn-MOF upon US irradiation. The Mn-MOF-induced antitumor immunity upon US irradiation might contribute to the tumor growth inhibition, and decreased tumor relapse and metastasis. Whether Mn-MOF-induced apoptosis upon US irradiation was also involved in the antitumor immunity, and the further biological application of Mn-MOF need to be further elucidated in the future research.

## Conclusions

In summary, we construct a versatile manganese porphyrin-based MOF (Mn-MOF) nanoplatform for enhanced SDT and ferroptosis. Mn-MOF exhibits high catalase-like activity that catalyzes tumor overexpressed H_2_O_2_ to generate O_2_ for tumor hypoxia relief. Meanwhile, Mn-MOF decreases intracellular GSH content and GPX4 activity. Thus, Mn-MOF shows enhanced SDT and ferroptosis to efficiently inhibit tumor growth and metastasis. In addition, Mn-MOF efficiently reshapes tumor immune microenvironment upon US irradiation by increasing the numbers of activated CD8^+^ T cells and matured DCs and decreaing the numbers of MDSCs in tumor tissues. Mn-MOF holds great potentials as an advanced system for achieving efficient hypoxic cancer treatment.

## Methods

### Materials

Pyrrole, 4-carbomethoxybenzaldehyde, propionic acid, MnCl_2_·4H_2_O, tetrahydrofuran (THF), ZrOCl_2_·8H_2_O, benzoic acid, DMF and 4'6-diamidino-2-phenylindole (DAPI) were purchased from Sinopharm Chemical Reagent Co. Ltd (Beijing, China). RPMI 1640 and fetal bovine serum (FBS) were purchased from Gibco RBL/Life Technologies (Grand Island, NY, USA). SOSG was obtained from Thermo Fisher Scientific Inc. (Waltham, MA, USA). 2'7'-DCFH-DA was purchased from Sigma-Aldrich Company (St Louis, MO, USA). GSH assay kit, GPX assay kit and LPO assay kit were purchased from Nanjing Jiancheng Bioengineering Institute (Nanjing, China). All other regents were of analytical grade and used without any further purification.

### Synthesis of Mn-MOF

Mn-MOF was synthesized by a solvothermal reaction between ZrOCl_2_·8H_2_O and Mn-TCPP 44. To synthesize Mn-TCPP 45, 4-carbomethoxybenzaldehyde (16.4 g, 0.1 mol) and pyrrole (6.9 mL, 0.1 mol) in 250 mL of propionic acid were refluxed and stirred for 2 h. The mixture solutions were then cooled to room temperature, and the resultant purple precipitates were filtered and washed with ethanol. The obtained H_2_TCPP-OMe was dissolved in CH_2_Cl_2_ and then purified by silica gel column (200-300 mesh) using CH_2_Cl_2_ and ethyl acetate (v/v, 100/1) as mobile phase. Furthermore, H_2_TCPP-OMe (0.42 g, 0.5 mmol) and MnCl_2_·4H_2_O (0.49 g, 2.48 mmol) in 50 mL of DMF was refluxed and stirred at 260 °C for 12 h. After cooling to room temperature, DMF was discarded by reduced pressure distillation. The obtained Mn-TCPP-OMe was washed with deionized water three times and dried. Mn-TCPP-OMe (0.44 g, 0.5 mmol) dissolved in 20 mL THF and KOH (1.12 g, 20 mmol) dissolved in 20 mL of deionized water were stirred at room temperature for 72 h. THF was discarded by reduced pressure distillation, and the resulting Mn-TCPP was treated with 2% HCl to adjust pH to 1-2 and washed with deionized water. TCPP was obtained using the same protocol except that TCPP-OMe did not react with MnCl_2_·4H_2_O. The chemical structures of TCPP and Mn-TCPP were determined by FT-IR spectrometer (Equinox55, Bruker, Germany), NMR spectrometer (600 MHz Bruker AscendTM, Bruker, Germany) and MALDI-TOF/TOF 5800 mass spectrometer (ABSciex, Framingham, MA, USA).

ZrOCl_2_·8H_2_O (30 mg, 0.093 mmol), benzoic acid (0.28 g, 2.29 mmol) and Mn-TCPP (10.98 mg, 0.013 mmol) were dissolved in 14 mL of DMF and the mixtures were stirred at 90 °C for 5 h. After the reaction, the mixtures were cooled to room temperature and centrifuged at 15, 000 rpm for 15 min. The pellets were washed with fresh DMF for three times and deionized water for three times. The resulting Mn-MOF was suspended in deionized water for further experiments. MOF was synthesized according to the same procedure except adding TCPP instead of Mn-TCPP.

### Characterization

The hydrodynamic diameter and zeta potential of MOF and Mn-MOF were determined using DLS (ZetaSizer ZS 90, Malvern Instruments Ltd., Worcestershire, UK). The morphology of MOF and Mn-MOF was observed by TEM (Tecnai G2-20, FEI, Netherlands). PXRD patterns of MOF and Mn-MOF were detected on a Bruker D8 Advance X-ray diffractometer (Bruker AXS GmbH, Karlsruhe, Germany) at a scanning rate of 4º/min with 2θ broadening from 3 to 30° by Cu Ka radiation. TGA of Mn-TCPP, MOF and Mn-MOF was performed on a PerkinElmer Pyris1 TGA (PerkinElmer Instruments, Waltham, MA, USA) from room temperature to 600 °C at a heating rate of 10 °C/min. The specific surface area and pore size of MOF and Mn-MOF were determined by the nitrogen adsorption technique at 150 °C on a micromeritics-ASAP 2420-4 system (Norcross, GA, USA). The elemental composition and valence state of Mn-MOF were evaluated by XPS (AXIS-ULTRA DLD-600W, Shimdzu, Japan). UV-Vis-NIR absorption spectra of MOF and Mn-MOF were conducted on a TU 1901 UV-Vis-NIR spectrometer (Beijing Persee, Beijing, China).

### Electrochemical measurement

Electrochemical analysis of Mn-MOF was performed on a CHI 660C electrochemical workstation (Chenhua Instrument, Shanghai, China) with a three-electrode electrochemical cell, which includes a working electrode, a platinum wire counter electrode and a KCl-saturated calomel reference electrode. The working electrode was prepared by dropping 5.0 μL of Mn-MOF dispersions at Mn concentration of 8 μg/mL on the glass carbon electrode (GCE) and drying in air. The electrochemical behaviors of the modified GCE in H_2_O_2_ at different concentrations were evaluated through CV. The scan range of CV was from 0.6 to 1.2 V with scan rate of 100 mV/s.

### Stopped-flow spectroscopy

The catalysis of H_2_O_2_ by Mn-MOF was determined using the SX20 stopped-flow fluorescence reaction rate analyzer (Applied phtotphysics Inc., Surrey, UK) equipped with a temperature controlled circulating water bath at 37 °C. Mn-MOF in PBS at Mn concentration of 8 μg/mL was placed in one syringe and 400 μM H_2_O_2_ was placed in another syringe. The syringes were rapidly mixed and the change of Mn-MOF absorbance as a function of time was recorded in the wavelength range from 440 nm to 500 nm.

### *In vitro* O_2_ generation

Mn-TCPP, MOF or Mn-MOF at different Mn concentrations (MOF was quantified according to the respective Zr concentration of Mn-MOF) was incubated with different concentrations of H_2_O_2_ at 37 °C. The dissolved O_2_ concentration was detected by a portable dissolved oxygen meter (JPSJ-605F, INESA & Scientific Instrument Co., LTD, Shanghai, China) in real time.

### GSH adsorption capacity

Mn-TCPP, MOF or Mn-MOF at Zr concentration of 10 and 20 μg/mL (the corresponding Mn concentration was 2 and 4 μg/mL for Mn-TCPP) was incubated with 5 μM GSH at 37 °C for 40 min. The samples were centrifuged at 15,000 rpm for 10 min and the supernatants were collected for GSH measurement by a GSH assay kit according to the manufacturer's guidance.

### *In vitro* ROS generation

Mn-TCPP, MOF or Mn-MOF was suspended in deionized water at Zr concentration of 50 µg/mL and Mn concentration of 10 µg/mL and then incubated with 400 µM H_2_O_2_. SOSG at the final concentration of 5 μM was added to the above suspensions and the mixtures were irradiated with US (1 MHz, 0.9 W/cm^2^, 30% duty cycle) for different time intervals under normoxic (air with about 21% O_2_) and hypoxic (a closed box filled with nitrogen gas) conditions. The fluorescence was measured by a Fluoromax-Plus spectrofluorometer (Horiba-Jobin Yvon Inc, Edison, NJ, USA) at the excitation and emission wavelength of 488 nm and 525 nm, respectively.

### Cells and animals

H22 cells and 4T1 cells were purchased from Type Culture Collection of Chinese Academy of Sciences (Shanghai, China). The cells were cultured in RPMI 1640 medium containing 10% FBS, 100 U/mL penicillin and 100 μg/mL streptomycin in a 5% CO_2_ incubator at 37 °C. BALB/c mice (male and femal, 6 weeks, 20-22 g) were provided by Beijing Vital River Laboratory Animal Technology Co., Ltd. (Beijing, China). H22 tumor-bearing mice were constructed by subcutaneous injection of 2×10^6^ H22 cells into the back of male BALB/c mice. 4T1 tumor-bearing mice were constructed by subcutaneous injection of 1×10^6^ 4T1 cells into the back of female BALB/c mice. All animal experiments were approved by the Institutional Animal Care and Use Committee at Tongji Medical College, Huazhong University of Science and Technology (Wuhan, China).

### Cellular uptake

H22 and 4T1 cells were seeded in a 12-well plate at a density of 1×10^5^ cells/well overnight. The cells were then treated with Mn-TCPP, MOF or Mn-MOF at Zr concentration of 10 μg/mL and Mn concentration of 2 μg/mL for different time intervals. The cells were washed with PBS three times and then the intracellular accumulation of TCPP was determined by flow cytometry and confocal microscope. For confocal microscopic analysis, the cells were stained with 2.5 μg/mL DAPI for 15 min and 50 nM LysoTracker green for 45 min at 37 °C and then observed by an Olympus FV1000 confocal microscope (Tokyo, Japan).

### Endocytic pathway

4T1 cells were seeded in a 12-well plate at a density of 1.5×10^5^ cells/well overnight. The cells were pretreated with EIPA (50 μM), CPZ (10 μg/mL) or mβCD (2 mM) for 1 h. The cells were then treated with MOF or Mn-MOF at Zr concentration of 10 μg/mL in the presence of the above inhibitors for another 2 h. The cells were washed with PBS three times and then the intracellular accumulation of TCPP was determined by flow cytometry.

### Intracellular ROS generation

H22 and 4T1 cells were seeded in a 12-well plate at a density of 1×10^5^ cells/well overnight under normoxic (21% O_2_) or hypoxic conditions overnight. For hypoxia groups, cells were cultured in a tri-gas incubator (Hua Xi Electronics Technetronic Co., Ltd, China) with a 2% oxygen concentration. The cells were then treated with Mn-TCPP, MOF or Mn-MOF at Zr concentration of 10 μg/mL and Mn concentration of 2 μg/mL for 10 h under normoxia or hypoxia. The cells were washed with PBS and then incubated with 5 μM 2'7'-DCFH-DA in dark for 30 min. The cells were irradiated with or without US (1 MHz, 0.9 W/cm^2^, 30% duty cycle) for 10 min under normoxia or hypoxia. After irradiation, the cells were washed with PBS three times and the intracellular ROS generation was determined by flow cytometry (FC500, Beckman Coulter, Fullerton, CA, USA) and Olympus FV1000 confocal microscope.

### Intracellular GSH content

H22 and 4T1 cells were seeded in a 12-well plate at a density of 1.5×10^5^ cells/well overnight. The cells were then treated with Mn-TCPP, MOF or Mn-MOF at different Zr concentrations under normoxia for 24 h. The cells were washed with PBS three times and then lysed by repeated cycles of freezing and thawing. The supernatants were collected for GSH measurement using a GSH kit according to the manufacture's protocol.

### GPX4 activity and LPO level

H22 cells were treated with Mn-TCPP, MOF or Mn-MOF at the Zr concentration of 10 μg/mL and Mn concentration of 2 μg/mL under normoxia for 10 h, followed with or without US irradiation (1 MHz, 0.9 W/cm^2^, 30% duty cycle) for 10 min. The cells were washed with PBS three times and then lysed by repeated cycles of freezing and thawing. The supernatants were collect, and the GPX4 activity and LPO levels were determined by glutathione peroxidase assay kit and LPO assay kit, respectively.

### *In vitro* cytotoxicity

H22 and 4T1 cells were seeded in a 96-well plate at a density of 1×10^4^ cells/well under normoxia (21% O_2_) or hypoxia (2% O_2_) overnight. The cells were then treated with Mn-TCPP, MOF or Mn-MOF at different Zr concentrations in the presence or absence of 10 nM UAMC-3203 for 10 h under normoxia or hypoxia at 37 °C. The cells were washed with PBS and then irradiated with or without US (1 MHz, 0.9 W/cm^2^, 30% duty cycle) for 10 min. The cells were further incubated for 24 h and the cell viability was determined using MTT assay. Briefly, the cells were washed with PBS and then incubated in the media containing 0.5 mg/mL MTT at 37 °C for another 4 h. The media were discarded and 150 μL DMSO was added to dissolve formazan crystals. The absorbance of formazan at 490 nm was measured by a Labsystems iEMS microplate reader (Helsinki, Finland).

### *In vivo* biodistribution

When the tumor volume of H22 tumor-bearing mice grew up to 100-150 mm^3^, the mice were randomly divided into three groups (n = 4 per group) and then intravenously injected with 0.1 mL of Mn-TCPP, MOF or Mn-MOF at Zr dosage of 5 mg/kg and Mn dosage of 1 mg/kg. At 24 h post-injection, the mice were sacrificed, and their major organs (heart, liver, spleen, lung and kidney) and tumors were collected and completely digested with Aqua Regia. Zr and Mn contents were determined by ICP-MS and expressed as percent of the injected Zr and Mn dose per gram of tissue.

### *In vivo* anticancer activity in H22 tumor-bearing mice

When the tumor volume of H22 tumor-bearing mice grew up to 100-150 mm^3^, the mice were randomly divided into eight groups (n = 13 per group) and then intravenously injected with PBS, Mn-TCPP, MOF or Mn-MOF at Zr dosage of 5 mg/kg and Mn dosage of 1 mg/kg in the presence or absence of intraperitoneal injection of UAMC-3203 at 20 μg/kg dosage. At 24 h after injection, the mice were or were not irradiated with US (1.0 MHz, 1 W/cm^2^, 50% duty cycle) for 10 min. The body weights were measured by using a scale-balance, and tumor lengths and widths were measured with a caliper every day. Tumor volume was calculated by the following equation: V= (tumor length)×(tumor width)^2^/2. On day 12 after treatment, 5 mice each group were sacrificed. Blood samples were collected for serological analysis. Tumors and other major organs (heart, live, spleen, lung and kidney) were harvested and fixed in a 4% paraformaldehyde for H&E staining. Tumor sections were stained using Cy3-conjugated anti-CD31 antibody and Cy3-conjugated anti-HIF-1α antibody. The rest of mice were used for long-term survival observation.

### *In vivo* anti-metastasis activity in 4T1 tumor-bearing mice

When the tumor volume of 4T1 tumor-bearing mice grew up to 100-150 mm^3^, the mice were randomly divided into eight groups (n = 13 per group) and then intravenously injected with PBS, Mn-TCPP, MOF or Mn-MOF at Zr dosage of 5 mg/kg and Mn dosage of 1 mg/kg. At 24 h after injection, the mice were or were not irradiated with US (1.0 MHz, 1 W/cm^2^, 50% duty cycle) for 5 min. The body weights were measured by using a scale-balance, and tumor lengths and widths were measured with a caliper every day. Tumor volume was calculated by the following equation: V= (tumor length)×(tumor width)^2^/2. On day 20 after treatment, 5 mice each group were sacrificed. Blood samples were collected for serological analysis. Lung tissues were weighed and the metastatic nodules on lungs were counted. Tumors and other major organs (heart, live, spleen, lung and kidney) were harvested and fixed in a 4% paraformaldehyde for H&E staining. Tumor sections were immunofluorescence stained using Cy3-conjugated anti-CD31 antibody, Cy3-conjugated anti-VEGF antibody and Cy3-conjugated anti-HIF-1α antibody. The rest mice were used for long-term survival observation.

### Tumor immune microenvironment analysis

When tumor volume of H22 tumor-bearing mice grew up to 100-150 mm^3^, the mice were intravenously injected with PBS, Mn-TCPP, MOF or Mn-MOF at Zr dosage of 5 mg/kg (the corresponding Mn dosage was 1 mg/kg) in the presence or absence of intraperitoneal injection of UAMC-3203 at 20 μg/kg dosage. At 24 h after injection, the tumors were irradiated with US irradiation (1.0 MHz, 1 W/cm^2^, 50% duty cycle) for 10 min. The tumor lengths and widths were measured with a caliper every day. On day 10 after treatment, the mice were sacrificed, and the tumors were cut into small fragments and digested in 5 mL RPMI 1640 media containing 0.8 mg/mL collagenase type I at 37 °C for 50 min. Single-cell suspensions were obtained using a 40 μm cell strainer and the cells were washed with Red Cell Lysis Solution. For surface marker analysis, the cells were stained with FITC anti-mouse CD3ε (Clone 145-2C11, 100306, Biolegend), APC/Cyanine7 anti-mouse CD45 (Clone 30-F11, 103116, Biolegend), PE/Cy7 anti-mouse CD8a (Clone 53-6.7, 100722, Biolegend), PerCP/Cyanine5.5 anti-mouse CD69 (Clone H1.2F3, 104522, Biolegend), FITC anti-mouse CD11c (Clone N418, 117305, Biolegend), PE anti-mouse CD80 (Clone 16-10A1, 104708, Biolegend), APC anti-mouse CD86 (Clone GL-1, 105012, Biolegend), FITC anti-mouse/human CD11b (Clone M1/70, 101206, Biolegend), APC anti-mouse Ly-6G/Ly-6C (Gr-1) (Clone RB6-8C5, 108412, Biolegend), PerCP/Cyanine5.5 anti-mouse CD4 (Clone RM4-5, 100540, Biolegend) or APC anti-mouse CD25 (Clone PC61, 102012, Biolegend) at 37 °C for 30 min. For intracellular cytokine staining, the cells were treated with Fix/Perm solution (420801, Biolegend) following by re-staining with PE anti-mouse IFN-γ (Clone XMG1.2, 505808, Biolegend) or APC anti-human/mouse Granzyme B recombinant antibody (Clone QA16A02, 372204, Biolegend). For transcription factor staining, cells were treated with the True-Nuclear™ Transcription Factor Buffer Set (424401, Biolegend) after surface staining and re-stained with PE anti-mouse FOXP3 (Clone MF-14, 126404, Biolegend). The cells were analyzed by the CytoFLEX S flow cytometry (Beckman coulter, Fullerton, CA, USA).

### Statistical analysis

All experiments were performed with at least three replicates. All values were presented as the mean values ± SD. Significance was evaluated by the Student's *t* test for comparison of 2 groups and one-way ANOVA for multiple groups. Values with *P <* 0.05 were considered significant.

## Supplementary Material

Supplementary figures and tables.
