# Supplementary material for: Manganese porphyrin-based metal-organic framework for synergistic sonodynamic therapy and ferroptosis in hypoxic tumors
Source: Theranostics. 2021 Jan 1;11(4):1937–52. doi: 10.7150/thno.45511 (PMC7778611; doi:10.7150/thno.45511)
Supplement: Supplementary file 1 — Supplementary figures and tables. [file thnov11p1937s1.pdf]

Supporting Information

Manganese Porphyrin-Based Metal-Organic Framework for Synergistic Sonodynamic Therapy and Ferroptosis in Hypoxic Tumors

Qingbo Xu[1,2,#], Guiting Zhan[1,#], Zelong Zhang[1], Tuying Yong[1], Xiangliang Yang[1,3,4,*], Lu Gan[1,3,4,*]

[1]National Engineering Research Center for Nanomedicine, College of Life Science and Technology, Huazhong University of Science and Technology, Wuhan 430074, China

[2]Zhuhai Precision Medical Center, Zhuhai People's Hospital (Zhuhai Hospital Affiliated with Jinan University), Zhuhai 519000, China

[3]Hubei Key Laboratory of Bioinorganic Chemistry and Materia Medica, School of Chemistry and Chemical Engineering, Huazhong University of Science and Technology, Wuhan 430074, China

[4]Key Laboratory of Molecular Biophysics of Ministry of Education, College of Life Science and Technology, Huazhong University of Science and Technology, Wuhan 430074, China

*Corresponding authors. *E-mail addresses*: lugan@mail.hust.edu.cn (L. Gan), yangxl@mail.hust.edu.cn (X. Yang)

[#]These authors contributed equally to this paper.

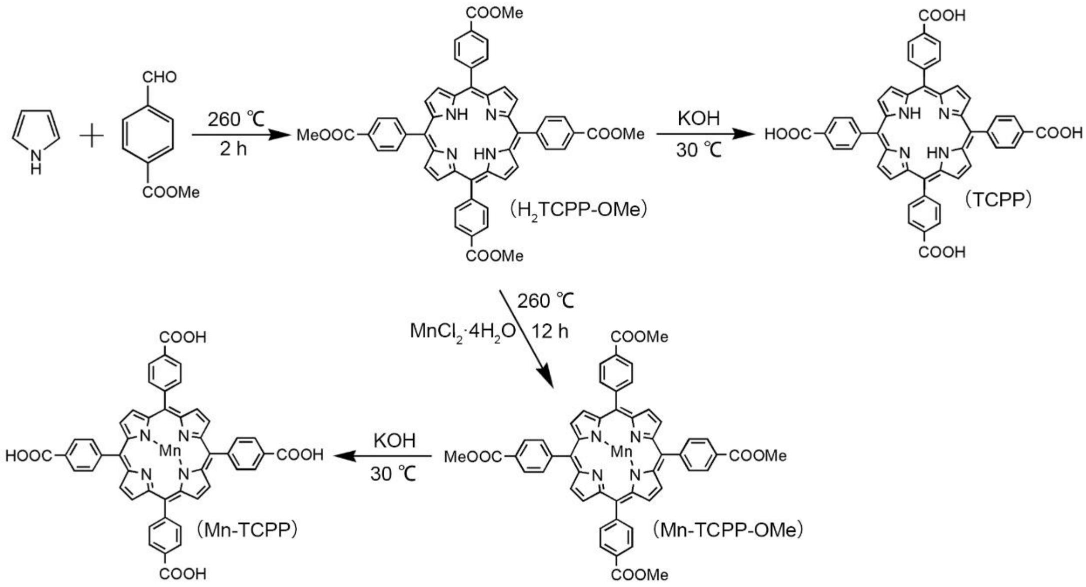

Supplementary Figure 1. Synthesis of Mn-TCPP and TCPP.

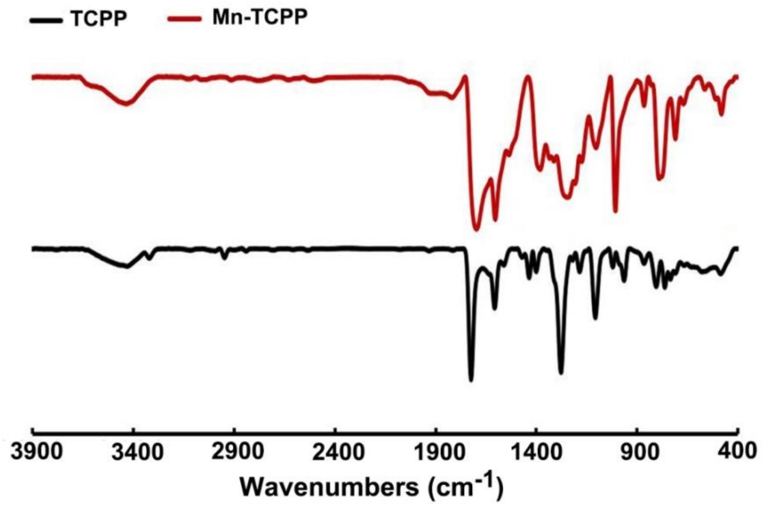

Supplementary Figure 2. FT-IR spectra of TCPP and Mn-TCPP.

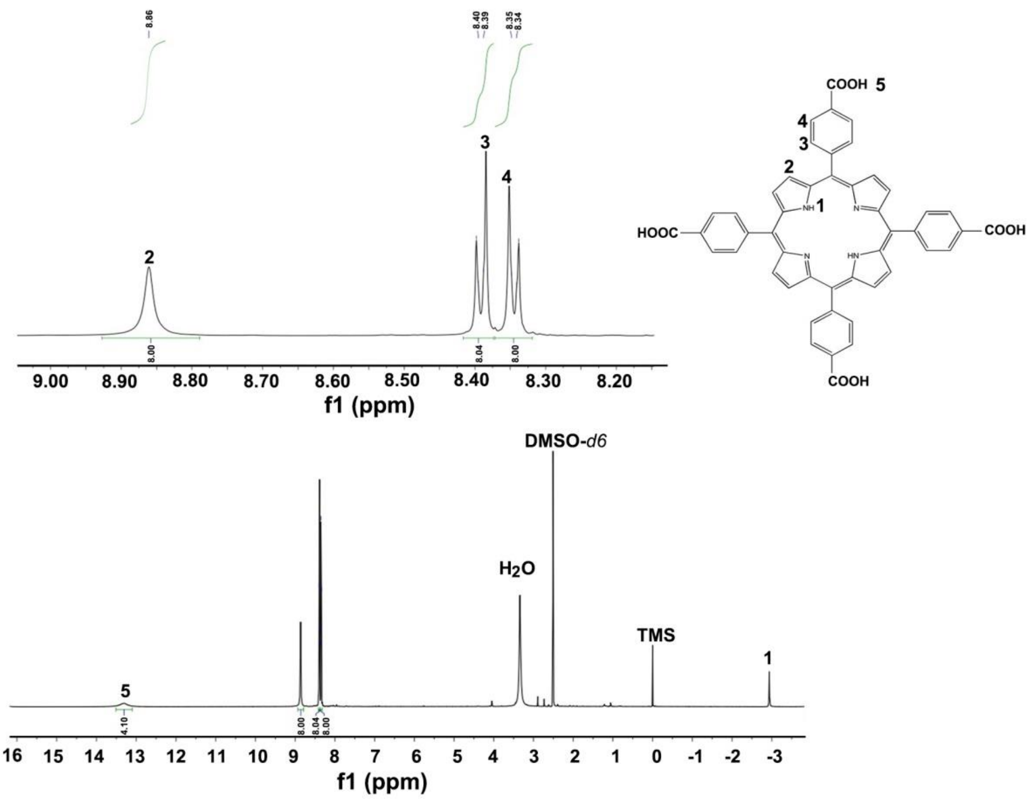

Supplementary Figure 3. ^{1}H NMR spectrum of TCPP. The protons 1-5 correspond to the peak position of H at -2.93 (2H,s), 8.86 (8H,s), 8.39 (8H,J=8.2 Hz, d), 8.34 (8H, J=8.1 Hz, d) and 13.28 (4H,s), respectively.

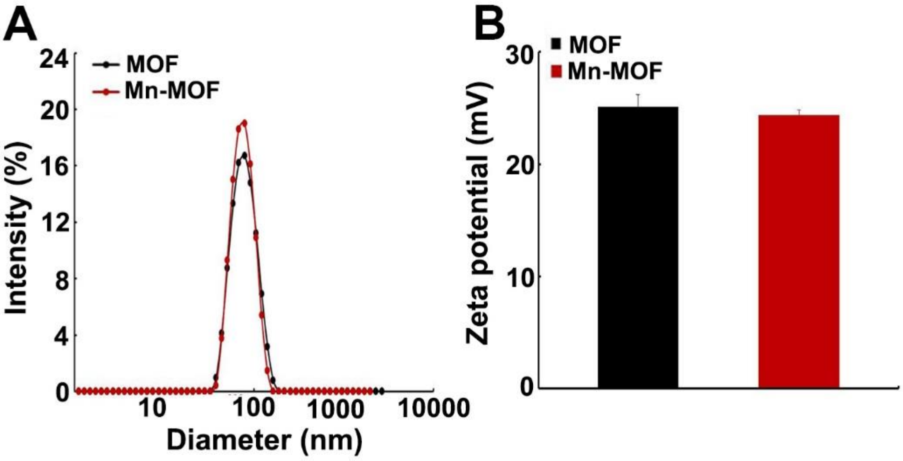

Supplementary Figure 4. The diameter (A) and zeta potential (B) of MOF and Mn-MOF by DLS analysis. The data are presented as mean ± s.d. (n= 3).

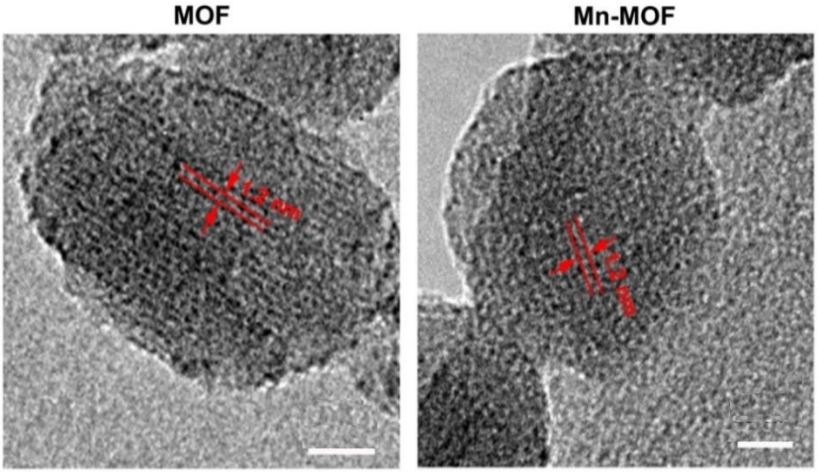

Supplementary Figure 5. High-resolution TEM images of MOF and Mn-MOF. Scale bar: 10 nm.

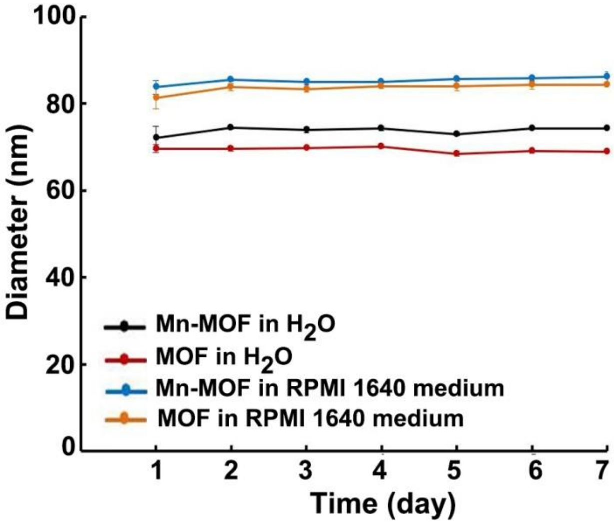

Supplementary Figure 6. Diameter of MOF and Mn-MOF after incubation in H_2O or RPMI 1640 medium for different time intervals. The data are presented as mean ± s.d. (n= 3).

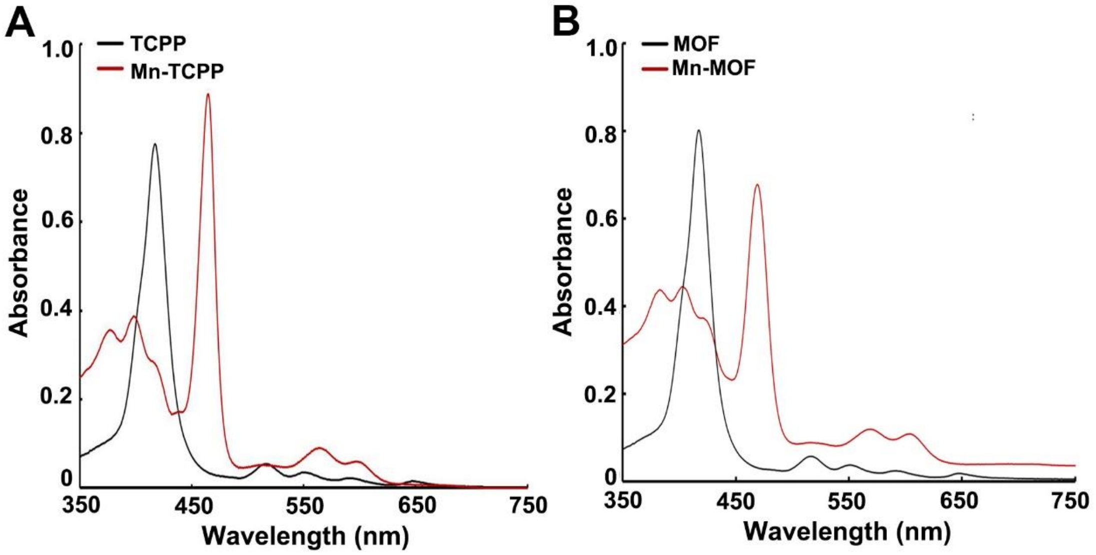

Supplementary Figure 7. UV-Vis absorption spectra of TCPP, Mn-TCPP (A), and MOF and Mn-MOF (B).

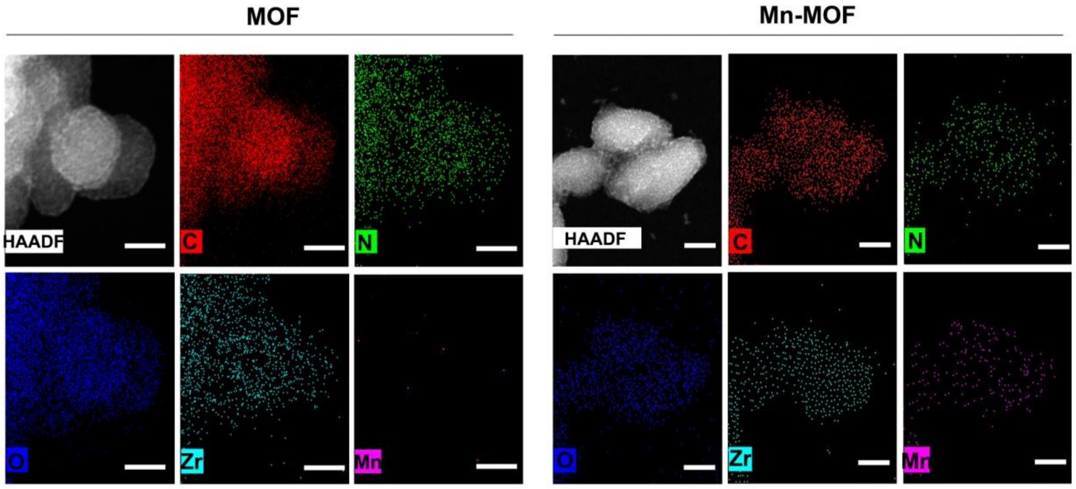

Supplementary Figure 8. Elemental mapping of MOF and Mn-MOF by EDX analysis. Scale bar: 20 nm.

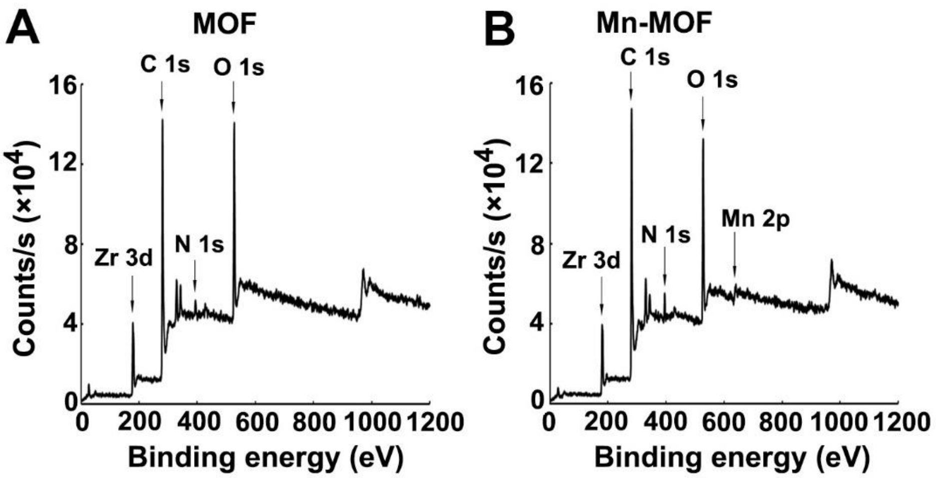

Supplementary Figure 9. XPS spectra of MOF (A) and Mn-MOF (B).

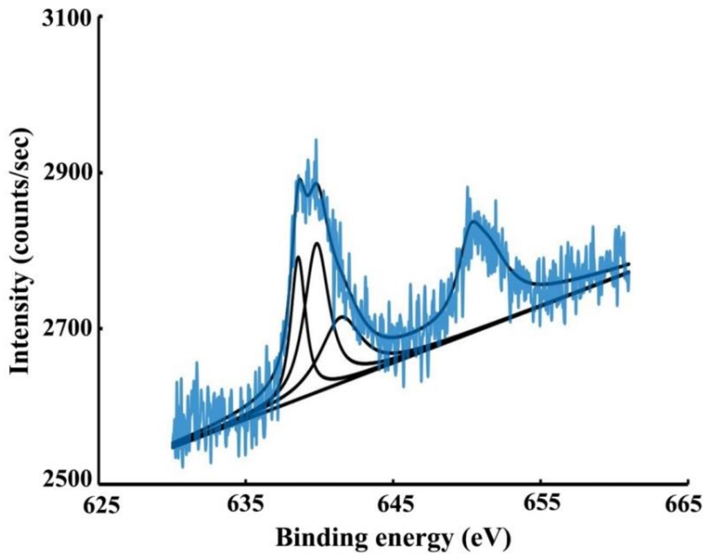

Supplementary Figure 10. Valence analysis of Mn in Mn-TCPP.

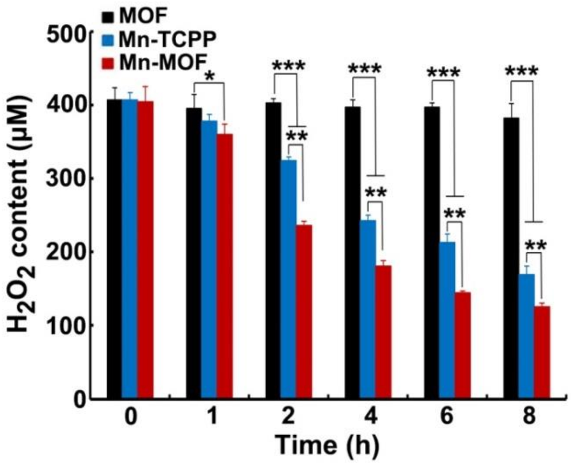

Supplementary Figure 11. H_2O_2 content after Mn-TCPP, MOF or Mn-MOF at Zr concentration of 5 μg/mL and Mn concentration of 1 μg/mL was incubated with 400 μM H_2O_2 for different time courses. The data are presented as mean ± s.d. (n= 3). *$P < 0.05$, **$P < 0.01$, ***$P < 0.001$.

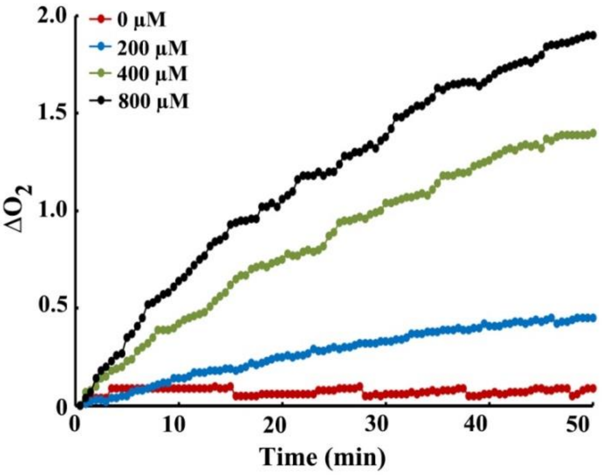

Supplementary Figure 12. O_2 generation after Mn-MOF at Mn concentration of 8 μg/mL was treated with different concentrations of H_2O_2 for different time intervals.

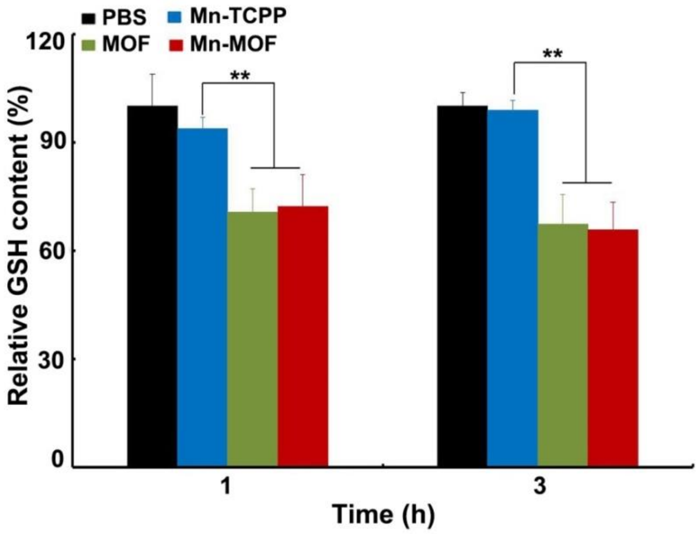

Supplementary Figure 13. GSH consumption after Mn-TCPP, MOF and Mn-MOF at the Zr concentration of 10 μg/mL (the corresponding Mn concentration is 2 μg/mL) were incubated with 5 mM GSH for different time intervals. The data are presented as mean ± s.d. (n=3). $^{**}P<0.01$.

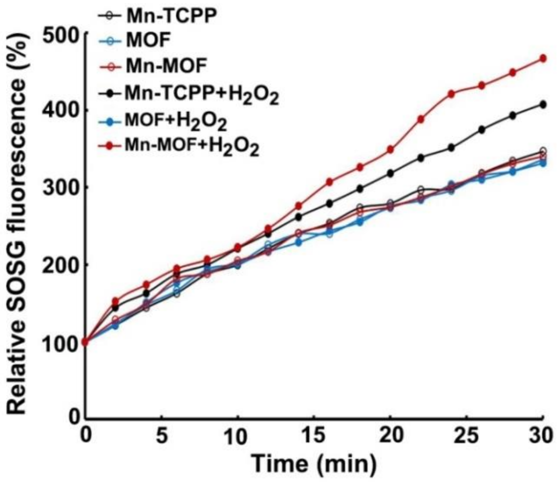

Supplementary Figure 14. ROS generation after Mn-TCPP, MOF and Mn-MOF at the Zr concentration of 50 µg/mL and Mn concentration of 10 µg/mL were treated with or without 400 µM H_2O_2 for different time intervals under normoxic conditions upon US irradiation (1 MHz, 0.9 W/cm^2, 30% duty cycle). The data are presented as mean ± s.d. (n= 3).

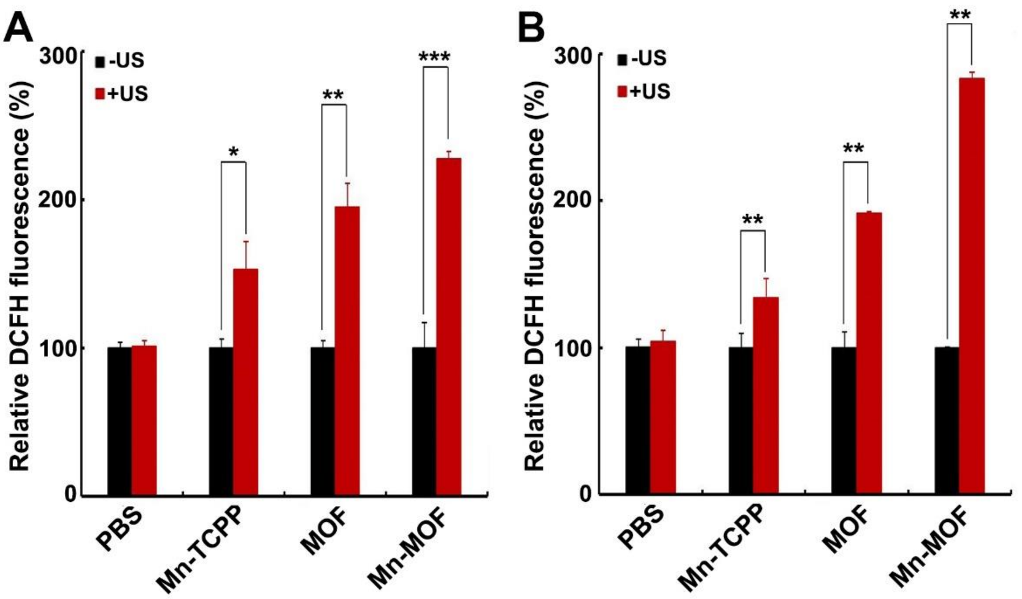

Supplementary Figure 15. Mn-MOF-induced intracellular ROS generation under normoxia in the presence or absence of US irradiation. (A,B) DCFH fluorescence in H22 (A) and 4T1 cells (B) treated with Mn-TCPP, MOF or Mn-MOF at the Zr concentration of 10 μg/mL and Mn concentration of 2 μg/mL for 10 h under normoxia in the presence or absence of US irradiation (1 MHz, 0.9 W/cm^2, 30% duty cycle). The data are presented as mean ± s.d. (n= 3). *P < 0.05, **P < 0.01, ***P < 0.001.

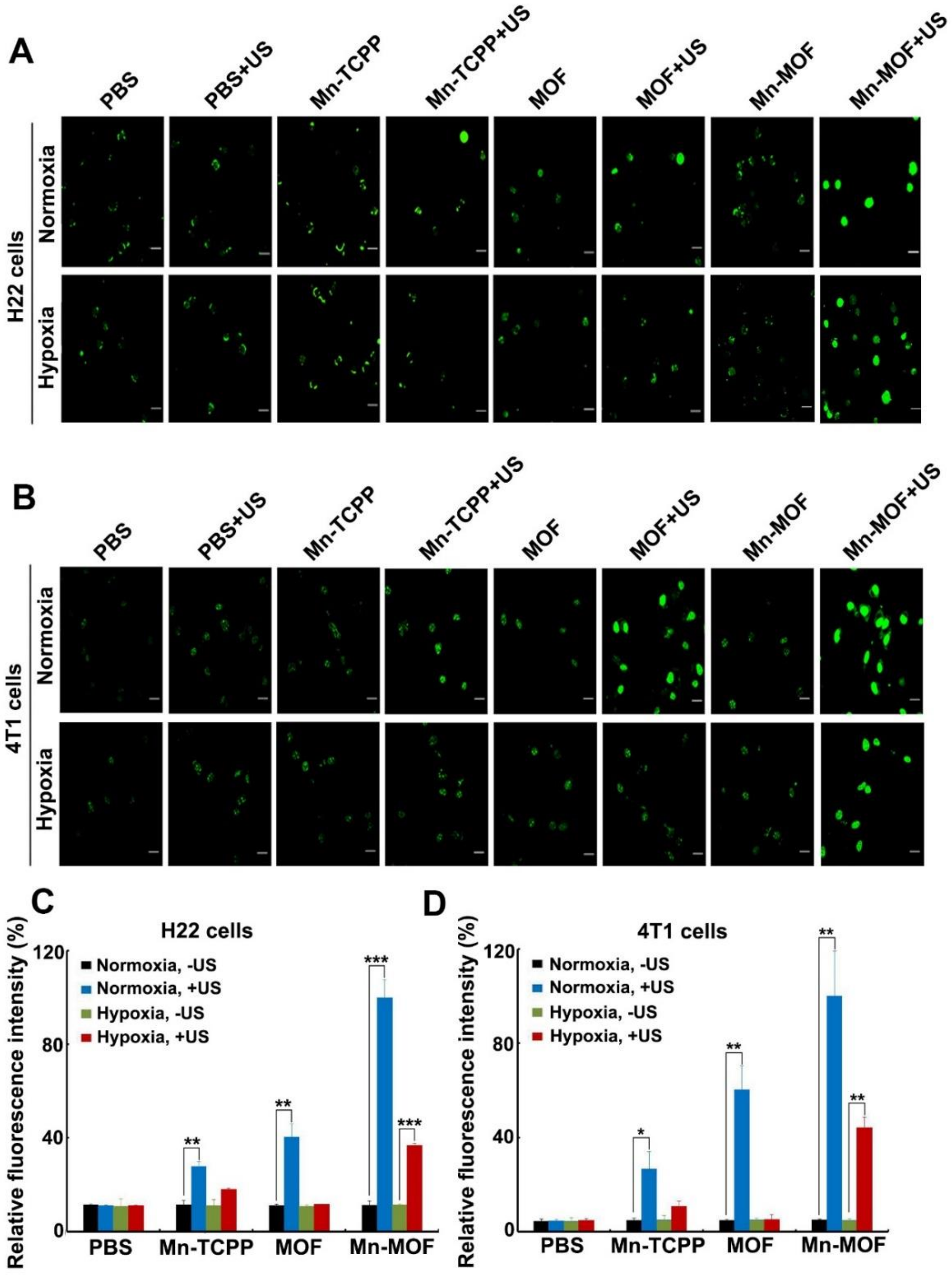

Supplementary Figure 16. Mn-MOF-induced intracellular ROS generation under normoxia or hypoxia in the presence or absence of US irradiation by confocal microscopy. (A,B) DCFH fluorescence in H22 (A) and 4T1 cells (B) treated with Mn-TCPP, MOF or Mn-MOF at the Zr concentration of 10 µg/mL and Mn concentration of 2 µg/mL for 10 h under normoxia or hypoxia in the presence or absence of US irradiation (1 MHz, 0.9 W/cm^2, 30% duty cycle) by confocal microscopy. Scale bar: 20 µm. (C,D) Relative DCFH fluorescence intensity in H22 (C) and 4T1 cells (D) as above. The data are presented as mean ± s.d. (n= 3). *P < 0.05, **P < 0.01, ***P < 0.001.

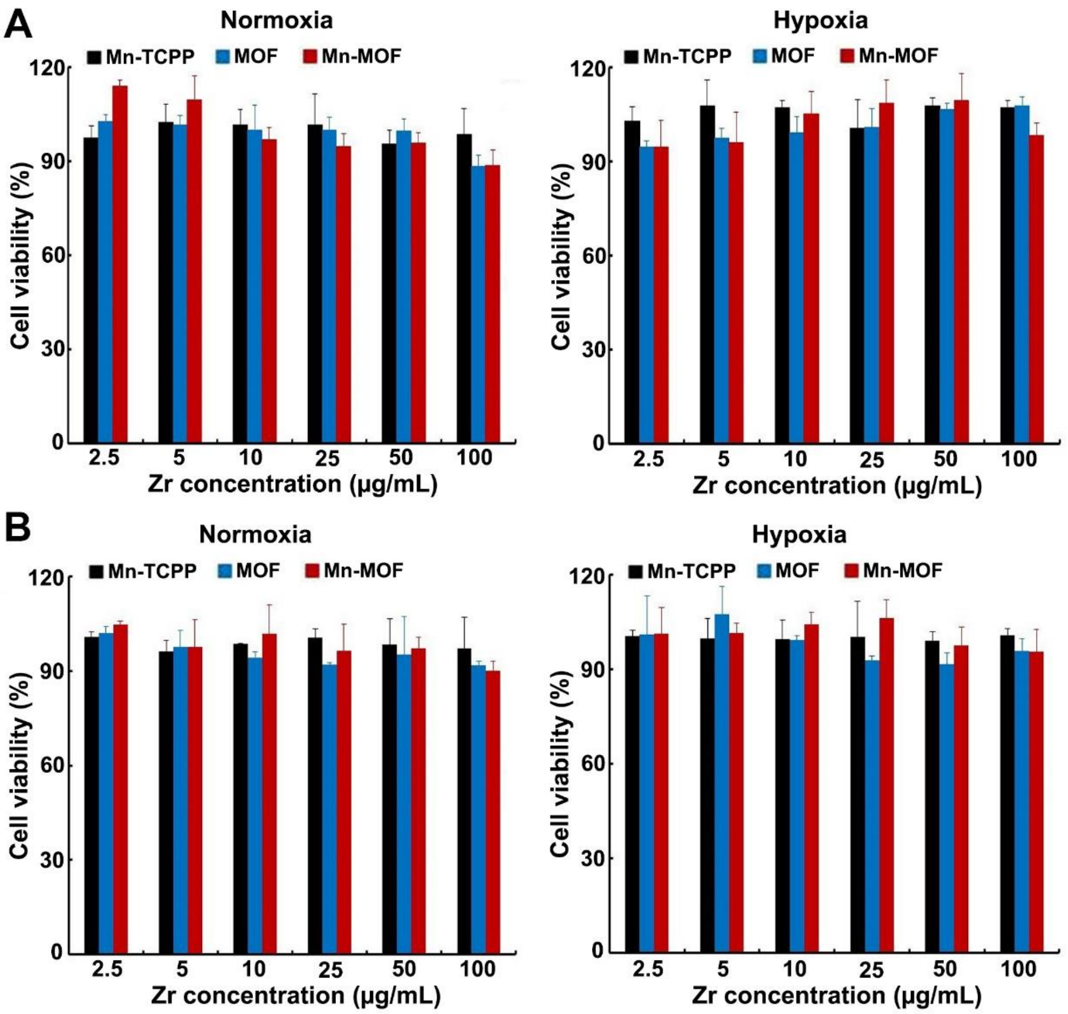

Supplementary Figure 17. The biocompatibility of Mn-MOF *in vitro*. (A,B) Cell viabilities of H22 (A) and 4T1 cells (B) after treatment with Mn-TCPP, MOF or Mn-MOF at the different Zr concentrations (The quantification of Mn-TCPP was calculated according to the corresponding Mn concentration of Mn-MOF) for 24 h under normoxia or hypoxia. The data are presented as mean ± s.d. (n= 3).

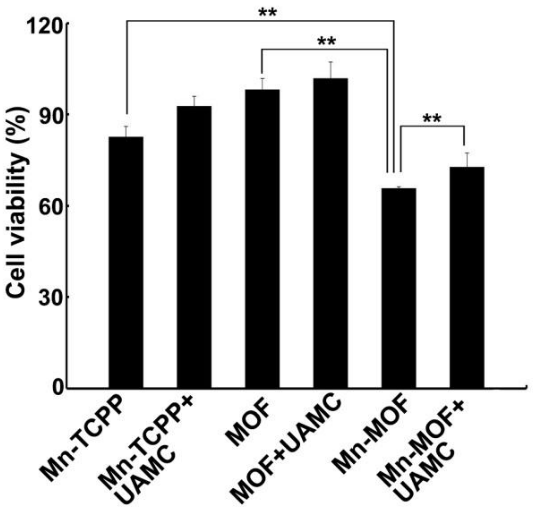

Supplementary Figure 18. Cell viability of H22 cells treated with Mn-TCPP, MOF or Mn-MOF at the Zr concentration of 4 μg/mL and Mn concentration of 0.8 μg/mL in the presence or absence of 10 nM UAMC-3203 for 10 h under hypoxia for 10 h, followed by US irradiation (1 MHz, 0.9 W/cm^2, 30% duty cycle) for 10 min. The data are presented as mean ± s.d. (n= 3). $^{**}P < 0.01$.

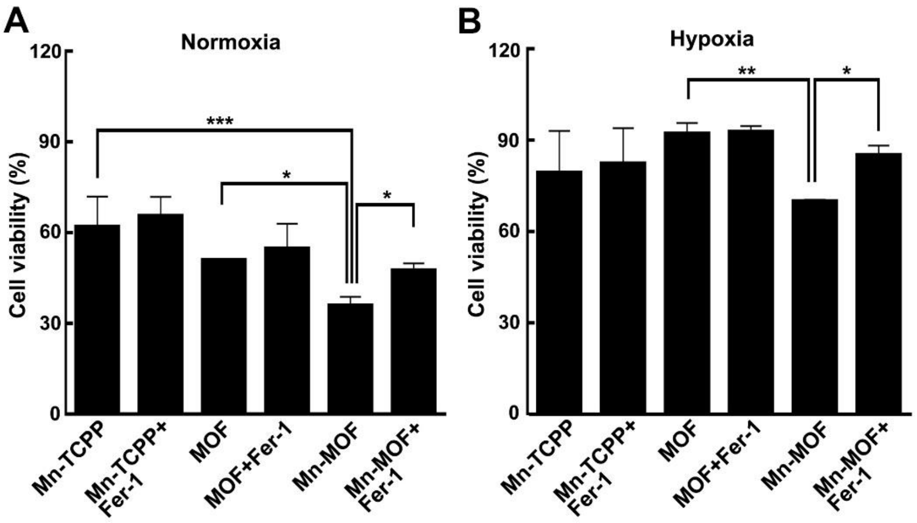

Supplementary Figure 19. Ferroptosis was involved in Mn-MOF-induced cell cytotoxicity upon US irradiation. Cell viability of H22 cells treated with Mn-TCPP, MOF or Mn-MOF at the Zr concentration of 4 μg/mL and Mn concentration of 0.8 μg/mL in the presence or absence of 1 μM Fer-1 for 10 h under normoxia (A) and hypoxia (B), followed by US irradiation (1 MHz, 0.9 W/cm^2, 30% duty cycle) for 10 min. The data are presented as mean ± s.d. (n= 3). ***P < 0.001, **P < 0.01, *P < 0.05.

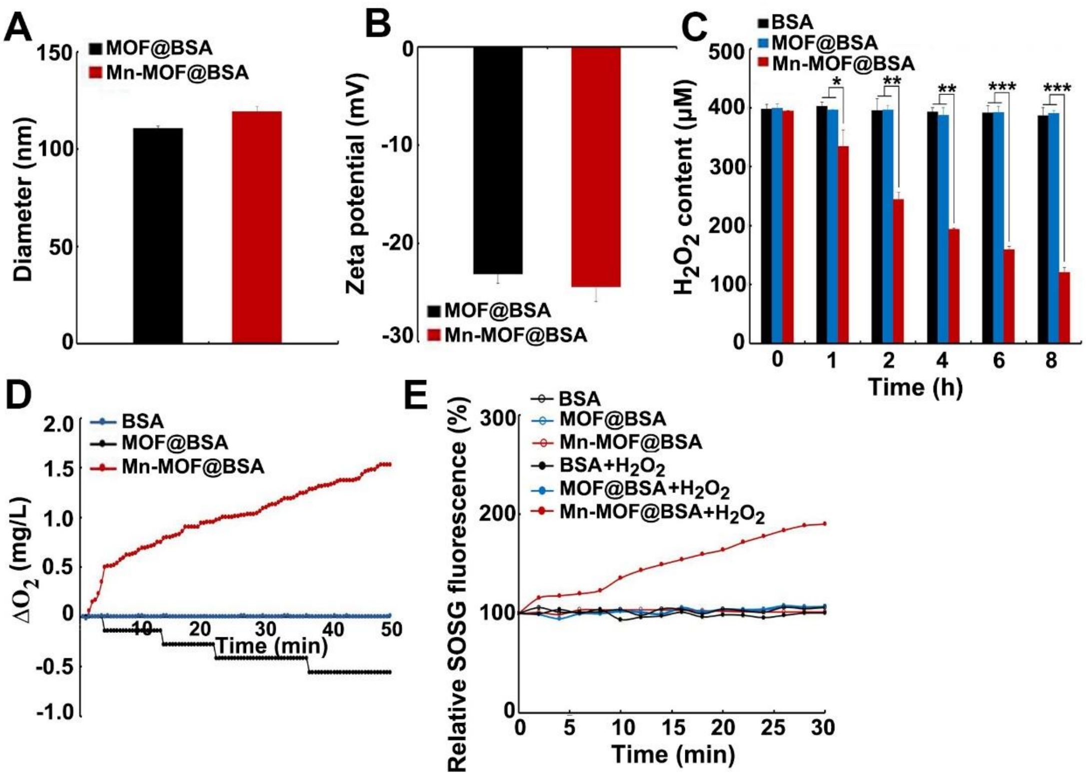

Supplementary Figure 20. Effects of BSA modification on the characteristics of Mn-MOF. (A,B) The diameter (A) and zeta potential (B) of MOF and Mn-MOF after BSA modification. (C) H_2O_2 content after BSA, MOF@BSA or Mn-MOF@BSA at Zr concentration of 5 μg/mL was incubated with 400 μM H_2O_2 for different time courses. (D) O_2 generation after BSA, MOF@BSA or Mn-MOF@BSA at the Zr concentration of 40 μg/mL and Mn concentration of 8 μg/mL were treated with 400 μM H_2O_2 for different time intervals. (E) ROS generation after BSA, MOF@BSA or Mn-MOF@BSA at the Zr concentration of 50 μg/mL were treated with or without 400 μM H_2O_2 for different time intervals under hypoxic conditions upon US irradiation (1 MHz, 0.9 W/cm^2, 30% duty cycle). The data are presented as mean ± s.d. (n= 3). **P* < 0.05, ***P* < 0.01, ****P* < 0.001.

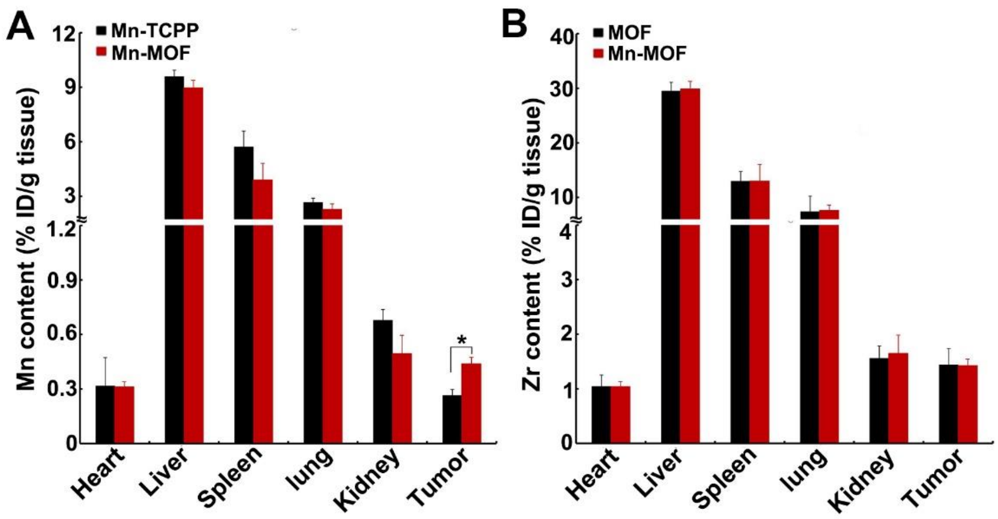

Supplementary Figure 21. The biodistribution of Mn-MOF in H22 tumor-bearing mice. (A) Mn contents in major organs and tumor tissues at 24 h after H22 tumor-bearing mice were intravenously injected with Mn-TCPP or Mn-MOF at Zr dosage of 5 mg/kg and Mn dosage of 1 mg/kg. (B) Zr contents in major organs and tumor tissues at 24 h after H22 tumor-bearing mice were intravenously injected with MOF or Mn-MOF at Zr dosage of 5 mg/kg and Mn dosage of 1 mg/kg. The data are presented as mean ± s.d. (n= 3). *$P < 0.05$.

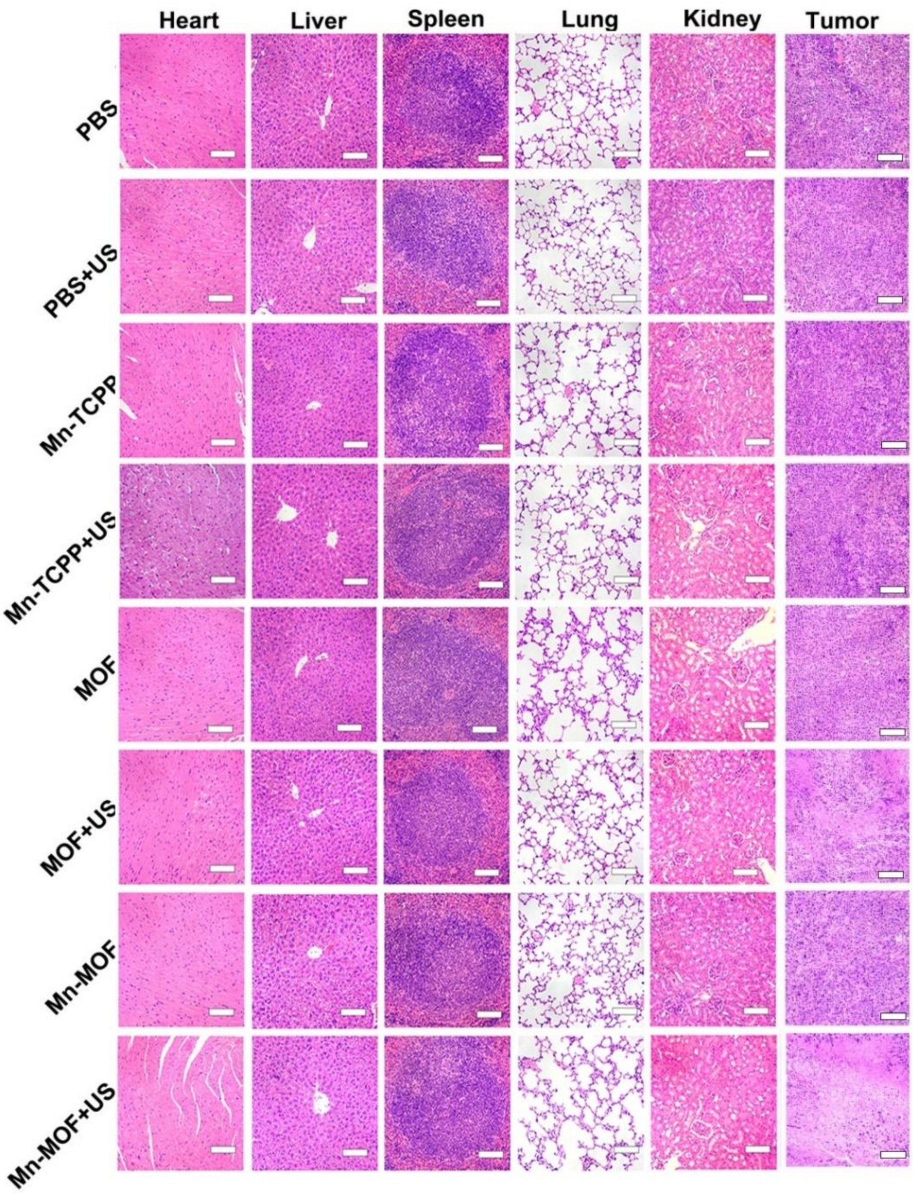

Supplementary Figure 22. Histological observation of tissues in H22 tumor-bearing mice after intravenous injection of PBS, Mn-TCPP, MOF or Mn-MOF at the Zr dosage of 5 mg/kg and Mn dosage of 1 mg/kg, followed by US irradiation (1 MHz, 1.0 W/cm^2, 50% duty cycle) for 10 min. Scale bar: 50 μm.

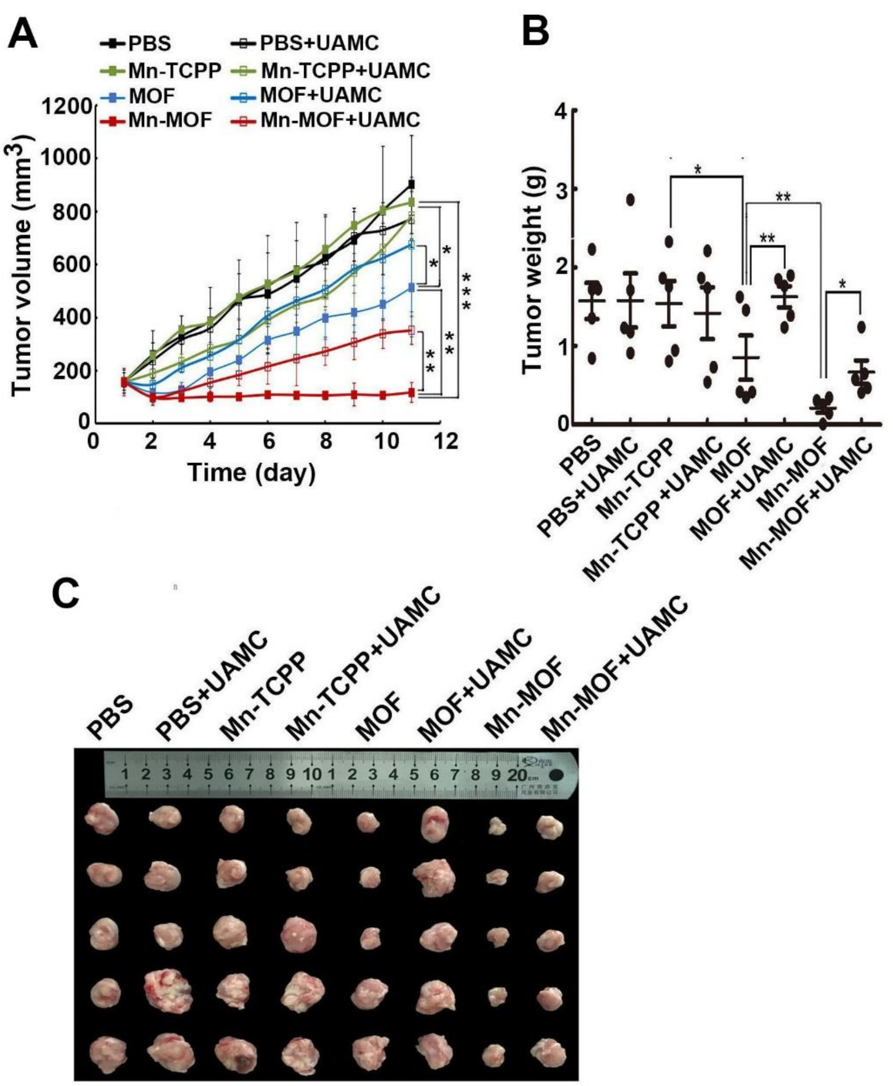

Supplementary Figure 23. Effects of UAMC-3203 on the Mn-MOF-induced anticancer activity in H22 tumor-bearing mice upon US irradiation. (A) Tumor volume of H22 tumor-bearing mice after intravenous injection of PBS, Mn-TCPP, MOF or Mn-MOF at Zr dosage of 5 mg/kg and Mn dosage of 1 mg/kg in the presence or absence of intraperitoneal injection of UAMC-3203 at 20 μg/kg dosage, followed by US irradiation (1.0 MHz, 1 W/cm^2, 50% duty cycle) at 24 h after injection. (B) Tumor weight of H22 tumor-bearing mice after treatment indicated in A. (C) Photographs of tumors of H22 tumor-bearing mice after treatment indicated in A. The data are presented as mean ± s.d. (n= 5). *$P < 0.05$, **$P < 0.01$, ***$P < 0.001$.

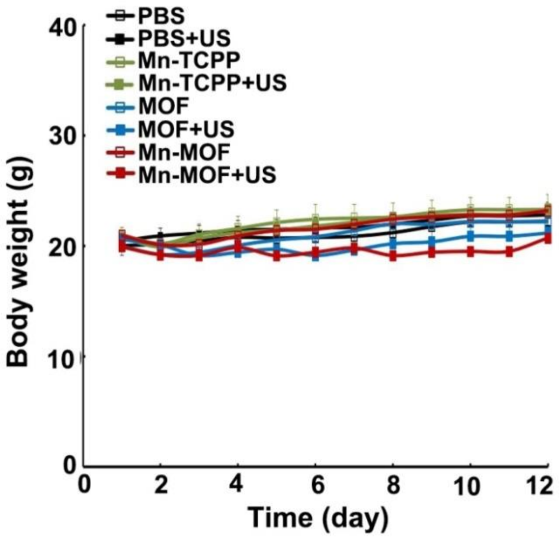

Supplementary Figure 24. Body weight of H22 tumor-bearing mice after intravenous injection of PBS, Mn-TCPP, MOF or Mn-MOF at the Zr dosage of 5 mg/kg and Mn dosage of 1 mg/kg, followed by US irradiation (1 MHz, 1.0 W/cm^2, 50% duty cycle) for 10 min at 24 h after injection. The data are presented as mean ± s.d. (n= 13).

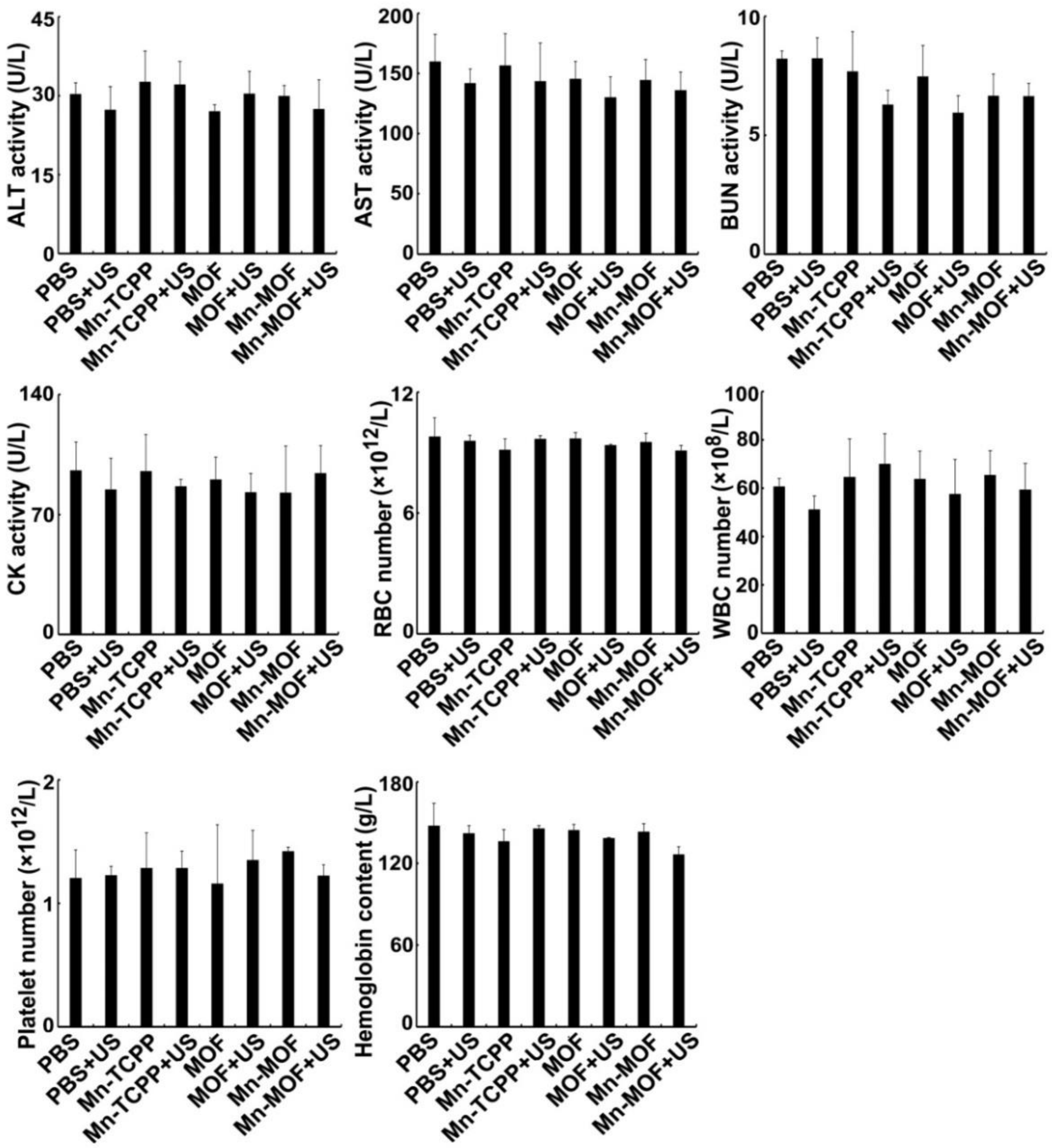

Supplementary Figure 25. Serological analysis of H22 tumor-bearing mice after intravenous injection of PBS, Mn-TCPP, MOF or Mn-MOF at the Zr dosage of 5 mg/kg and Mn dosage of 1 mg/kg, followed by US irradiation (1 MHz, 1.0 W/cm^2, 50% duty cycle) for 10 min. The data are presented as mean ± s.d. (n= 3).

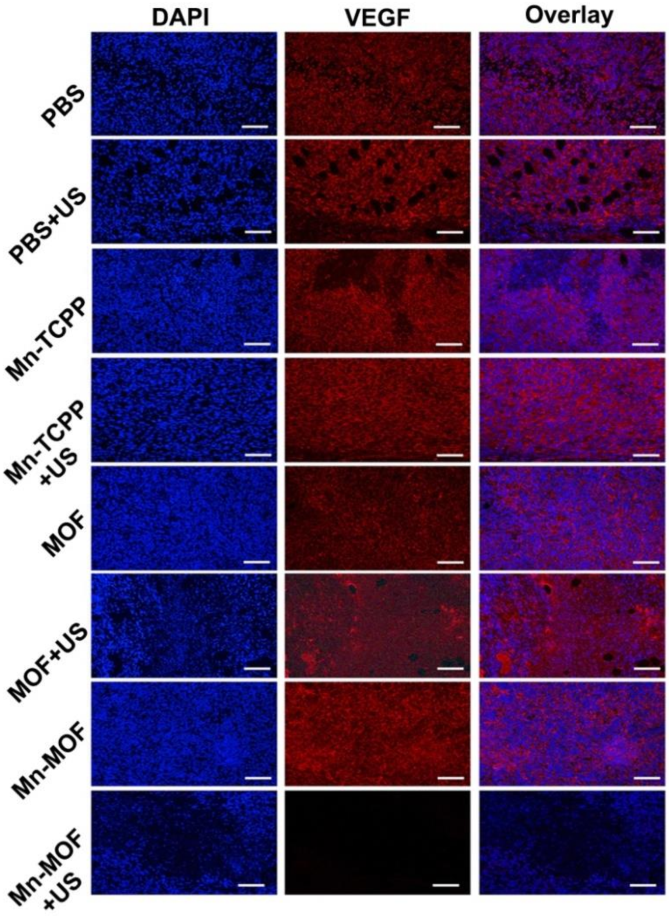

Supplementary Figure 26. Immunofluorescent staining of VEGF in tumor tissues of 4T1 tumor-bearing mice after intravenous injection of PBS, Mn-TCPP, MOF or Mn-MOF at the Zr dosage of 5 mg/kg and Mn dosage of 1 mg/kg, followed by US irradiation (1 MHz, 1.0 W/cm^2, 50% duty cycle) for 5 min. Scale bar: 50 μm.

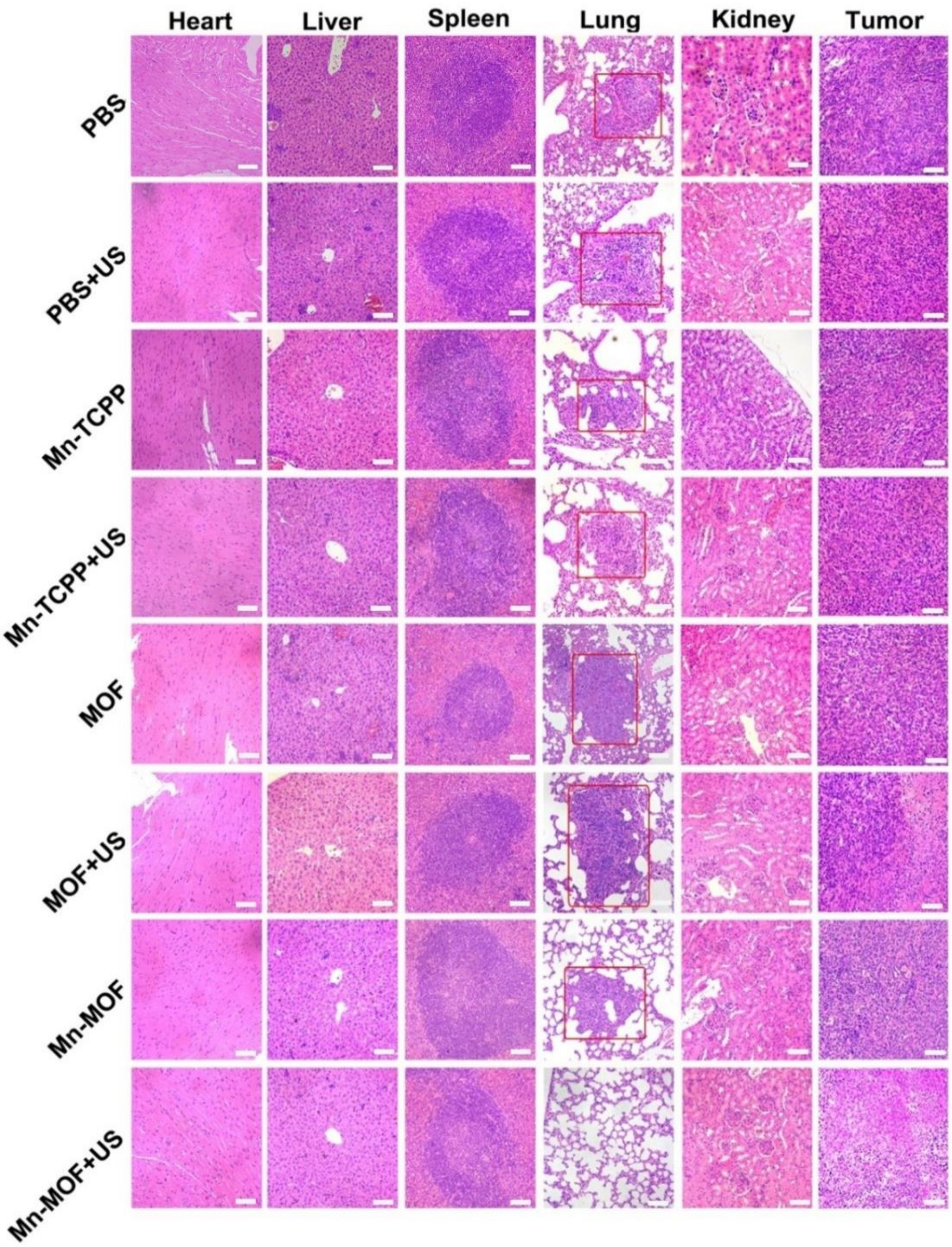

Supplementary Figure 27. Histological observation of tissues in 4T1 tumor-bearing mice after intravenous injection of PBS, Mn-TCPP, MOF or Mn-MOF at the Zr dosage of 5 mg/kg and Mn dosage of 1 mg/kg, followed by US irradiation (1 MHz, 1.0 W/cm^2, 50% duty cycle) for 5 min. Scale bar: 50 μm.

Supplementary Table 1. IC_{50} values of Mn-TCPP, MOF and Mn-MOF in H22 and 4T1 cells after treated with different formulations at the different Zr concentrations (the quantification of Mn-TCPP was calculated according to the corresponding Mn concentration of Mn-MOF) for 10 h under normoxia or hypoxia, followed by US irradiation (1 MHz, 0.9 W/cm^2, 30% duty cycle) for 10 min.

	H22 cells (μg Zr/mL)			4T cells (μg Zr/mL)		
	Mn-TCPP	MOF	Mn-MOF	Mn-TCPP	MOF	Mn-MOF
normoxia	13.75	4.917	3.556	7.449	4.738	3.739
hypoxia	n.d.	n.d.	10.4	n.d.	n.d.	14.25

n.d.: not determined.
